# Optogenetic β cell interrogation in vivo reveals a functional hierarchy directing the Ca^2+^ response to glucose supported by vitamin B6

**DOI:** 10.1126/sciadv.ado4513

**Published:** 2024-06-26

**Authors:** Luis Fernando Delgadillo-Silva, Emirhan Tasöz, Sumeet Pal Singh, Prateek Chawla, Eleni Georgiadou, Anne Gompf, Guy A. Rutter, Nikolay Ninov

**Affiliations:** ^1^ Centre for Regenerative Therapies TU Dresden, Dresden 01307, Germany.; ^2^ Paul Langerhans Institute Dresden of the Helmholtz Center Munich at the University Hospital Carl Gustav Carus of TU Dresden, German Center for Diabetes Research (DZD e.V.), Dresden 01307, Germany.; ^3^ Cardiometabolic Axis, CR-CHUM, and University of Montreal, Montreal, QC, Canada; 1IRIBHM, Université Libre de Bruxelles (ULB), 1070 Brussels, Belgium.; ^4^ IRIBHM, Université Libre de Bruxelles (ULB), Brussels, Belgium.; ^5^ Section of Cell Biology and Functional Genomics, Division of Diabetes, Endocrinology and Metabolism, Faculty of Medicine, Department of Metabolism, Digestion and Reproduction, Faculty of Medicine, Imperial College London, Hammersmith Hospital, London W12 ONN, UK.; ^6^ Lee Kong Chian School of Medicine, Nanyang Technological College, Singapore, Singapore.

## Abstract

Coordination of cellular activity through Ca^2+^ enables β cells to secrete precise quantities of insulin. To explore how the Ca^2+^ response is orchestrated in space and time, we implement optogenetic systems to probe the role of individual β cells in the glucose response. By targeted β cell activation/inactivation in zebrafish, we reveal a hierarchy of cells, each with a different level of influence over islet-wide Ca^2+^ dynamics. First-responder β cells lie at the top of the hierarchy, essential for initiating the first-phase Ca^2+^ response. Silencing first responders impairs the Ca^2+^ response to glucose. Conversely, selective activation of first responders demonstrates their increased capability to raise pan-islet Ca^2+^ levels compared to followers. By photolabeling and transcriptionally profiling β cells that differ in their thresholds to a glucose-stimulated Ca^2+^ response, we highlight vitamin B6 production as a signature pathway of first responders. We further define an evolutionarily conserved requirement for vitamin B6 in enabling the Ca^2+^ response to glucose in mammalian systems.

## Teaser

Optogenetic systems provide insights into the division of labor and the importance of cell heterogeneity for islet function.

## INTRODUCTION

Pancreatic β cells sustain glucose homeostasis by secreting precise amounts of insulin in proportion to energy-rich nutrients such as sugars. Insulin secretion in response to a stepped increase in glucose involves two phases (*1*). During the first phase, β cell depolarization triggers a rapid and transient increase in intracellular Ca^2+^ and the release of insulin. During the second phase, insulin is released in a more sustained and pulsatile manner. People with prediabetes or impaired glucose tolerance have a diminished first phase of secretion, while those with established type 2 diabetes (T2D) lose the first phase and show reduced second-phase insulin secretion (*2*). The pulsatility of secretion is also lost in T2D (*3*). Hence, it is of critical importance to understand the mechanisms of coordinated insulin secretion to develop effective therapies for diabetes.

Because pancreatic β cells undergo a synchronized influx of Ca^2+^ in response to glucose, they are often considered as a functional syncytium in which all of the cells work as a unit (*4*, *5*). Pioneering work from the Benninger (*6*) as well as the Stožer and Rupnik (*7*) groups reported intercellular heterogeneity during the first and second phases and also demonstrated the importance of gap-junction coupling and sugar metabolism for β cell synchronization. These findings were later extended to the human islet by the Rutter group (*8*). Using isolated mouse islets, Ca^2+^ imaging, and optogenetics, Westacott *et al*., Johnston *et al.,* and Stožer *et al.* further identified a subpopulation of highly connected β cells (“hubs”) that serve as possible pacemakers and regulate the Ca^2+^ waves during the oscillatory second phase of insulin release (*6*, *7*, *9*). Subsequently, leader cells, which initiate repeated Ca^2+^ waves during the second phase, were also identified and examined in mouse islets engrafted into the anterior chamber (*6*, *10*, *11*). In parallel, we have defined a critical subpopulation of β cells, which lead the Ca^2+^ increase during the first-phase response in zebrafish (*11*). These cells are referred to here as “first responders,” according to the recent terminology proposed by Kravets *et al.* and Stožer *et al.* in mouse islets (*12*, *13*). The laser-ablation of first responders in zebrafish can cause a decrease in the ability of the β cell population to mount an effective Ca^2+^ response to glucose (*11*). Recently, by analogous laser-cell ablation experiments of first responders in cultured mouse islets, Kravets *et al.* confirmed the importance of first responders for the first-phase response (*12*) and strengthened the view that the control over islet function is highly conserved across vertebrates. Nevertheless, the existence of controlling β cells and their properties has become the focus of intense investigation using both mouse and human islets (*14*,*15*) and has generated significant debates in the field, as reviewed recently (*16*–*19*). Therefore, the analysis of β cells in their natural environment using intravital microscopy in zebrafish and mice could help to address the outstanding questions about the functional heterogeneity of β cells and the underlying molecular differences.

While the zebrafish islet differs in its smaller size and relative simplicity of Ca^2+^ oscillations compared to mouse and human islets (*11*), the in vivo environment provides significant advantages when recording β cell activity where normal blood flow, innervation, and paracrine interactions are all preserved. Upon blood glucose infusion, zebrafish exhibit a very prominent and synchronized initial Ca^2+^ response, yet they show fewer secondary Ca^2+^ oscillations that are otherwise observable in mouse islets cultured under persistently high glucose (*11*, *20*). A recent study, however, has shown that mouse islets imaged intravitally in the anterior chamber of the eye exhibit a Ca^2+^ response to glucose with a prominent first-phase response but lacking marked secondary oscillations, as long as persistent hyperglycemia is avoided, akin to the situation in zebrafish (*21*).

The invasive approaches for studying β cell connectivity, such as irreversible cell ablation, have limited capability to interrogate the connectivity of multiple cells in the same tissue. To overcome this challenge, here, we deploy optogenetic tools, allowing us to depolarize or hyperpolarize individual cells in vivo in the zebrafish while assessing the impact on the first-phase Ca^2+^ response. In addition, we develop transgenic zebrafish lines enabling us to stably mark β cells that differ in the threshold to glucose-stimulated Ca^2+^ influx and, hence, to probe their gene expression differences. We find that first-responder cells represent a functionally and molecularly distinct group of β cells that control islet activity via the initiation of the first-phase Ca^2+^ response to elevated glucose. Across species, we identify a subpopulation of β cells expressing *pyridoxamine 5′-phosphate oxidase* (*pnpo*), a rate-limiting enzyme involved in vitamin B6 production, and show that this subpopulation coincides with the first-responder β cells in vivo. Using pharmacology and genetics, we reveal that *pnpo* and the vitamin B6 pathway support the coordinated Ca^2+^ response of β cells to glucose in zebrafish and mouse islets.

## RESULTS

### A proximity relationship between first-responder and follower cells directs the Ca^2+^ response in vivo

**Fig. 1.**
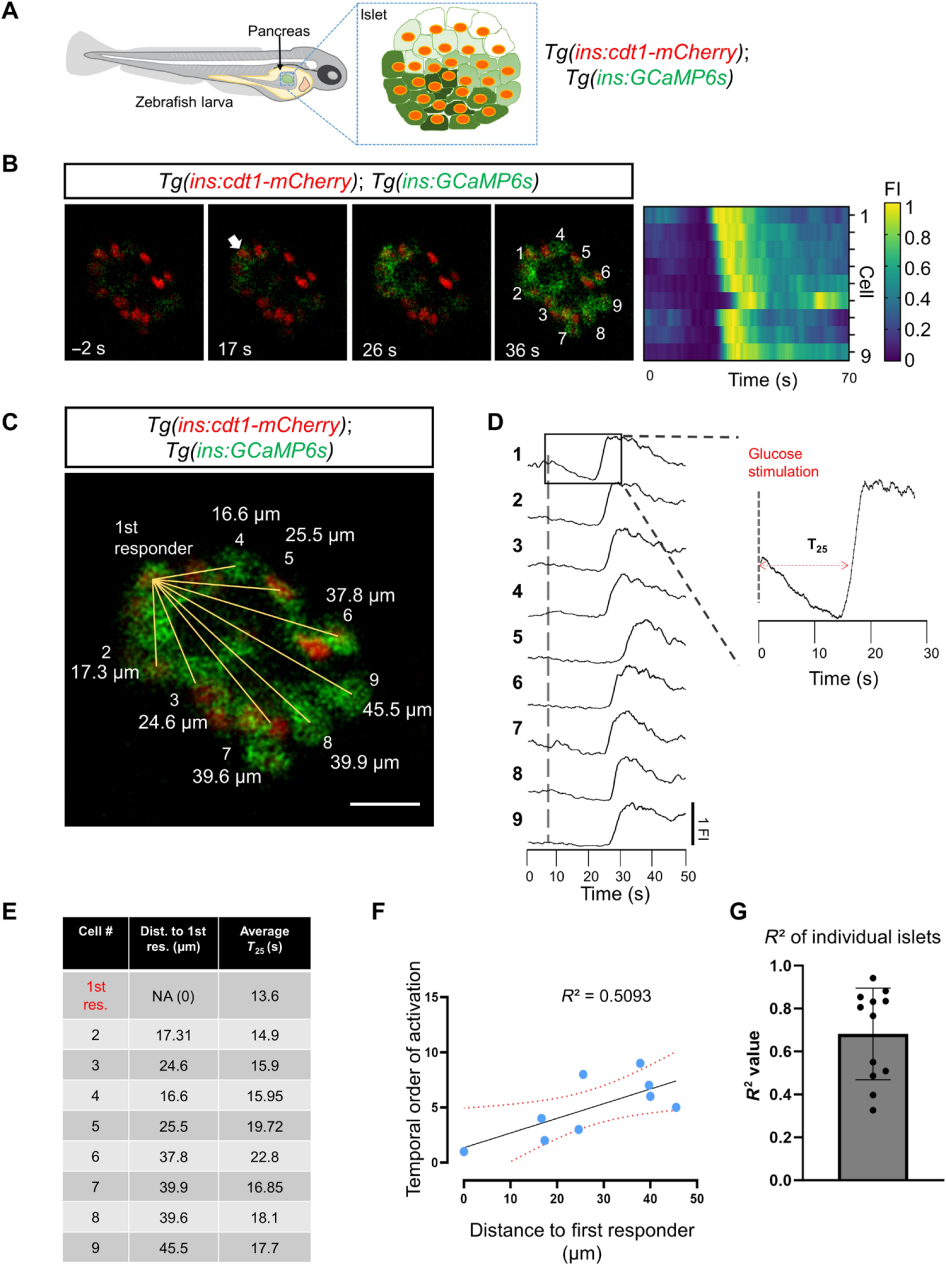
Calcium imaging in vivo reveals a proximity relationship between first-responder and follower cells. (**A**) Schematic representing a transgenic zebrafish expressing the genetically encoded calcium indicator GCaMP6 (green) and the nuclear marker cdt1-mCherry (red) in β cells. The insulin promoter expresses GCaMP6 in β cells, while cdt1-mCherry specifically labels the nuclei of β cells. (**B**) Snapshots from time-lapse recordings and raster plots of the primary islet at 6 Hz. The time stamp indicates the relative time to the glucose injection. The white arrow points to the β cell that responded first to the glucose stimulus. (**C**) The distance measured from the nuclei of the first-responder cell to the rest of the cells shown in (B). Scale bar, 10 μm. (**D**) Individual fluorescent traces from the cells shown in (B). (**E**) The table shows the distance (in μm) to the first-responder cell and the individual time of response (defined as a >25% increase in GCaMP6 fluorescence after glucose injection, *T*_25_). (**F**) Graph plotting the temporal order of activation of each cell versus the distance to the first responder from the islet shown in (B). The dotted line shows a linear regression and the associated *R*^2^ value. Each dot represents one cell. (**G**) The average *R*^2^ value was 0.609 with a ±SD of 0.27 (*n* = 12 independent samples). Data are shown as means ± SD. Cartoon of zebrafish larva in (A) reproduced with explicit permission from Nature Metabolism (www.nature.com/natmetab/)

To characterize the pattern of glucose-stimulated Ca^2+^ response in vivo, we used double transgenic *Tg(ins:GCaMP6s); Tg(ins:cdt1-mCherry)* larvae and studied their glucose responsiveness by performing calcium imaging (Fig. 1A). The *ins* promoter drives specific expression of genetically encoded calcium indicator 6s (GCaMP6s) in the zebrafish β cell. Upon glucose injection into larvae, all cells exhibit an increase in GCAMP6s fluorescence (Fig. 1B). The first-responder cell is the cell with the fastest average Ca^2+^ response from three consecutive stimulations with glucose, whereas the follower cells are the ones with slower responses (see Methods) (Fig. 1, C to E). Upon glucose stimulation, we found that the cumulative time of response for all β cells was 7.62 s after glucose injection (±6.05 s SD, *n* = 12 islets/larvae) (assessed as the time taken to achieve a GCaMP6 signal >25% above baseline, *T*_25_) (Fig. 1D). Plotting the relative speed of response of each cell as a function of its distance to the first-responder cell revealed a positive correlation between the time of response of each cell and its distance to the first-responder β cell coefficient of determination (*R*^2^) = 0.57 (± 0.24, *n* = 12 islets) (Fig. 1, F and G, and movie S1). These findings show that a proximity relationship between first-responder and follower cells underlies the Ca^2+^ response to glucose.

### Optogenetic silencing of single β cells can inhibit the islet responses to glucose

To be able to perform reversible and repeated interrogation of multiple β cells and assess the impact on intracellular Ca^2+^, we constructed several transgenic lines with optogenetic actuators with different optical spectra and capabilities (table S1) (*22*–*26*). The Halorhodopsin from the archaea *Natronomonas* (NpHR) is a light-gated chloride pump, which is activated by green light (λ = 560 to 590 nm) and can inhibit cell depolarization robustly (*25*). In principle, activating NpHR during a glucose stimulus should inhibit the glucose-induced influx of Ca^2+^ as a result of the electrical silencing of β cells. To test whether this approach can be applied in vivo in the fish setting, we generated transgenic larvae expressing eNpHR3.0-mCherry in β cells (*26*). Notably, we were able to temporally inhibit the glucose-induced influx of Ca^2+^, as reported by a reduction in the GCaMP signal (fig. S1 and movie S2), indicating that NpHR excitation effectively blocks depolarization and Ca^2+^ influx even in the presence of high glucose.

**Fig. 2.**
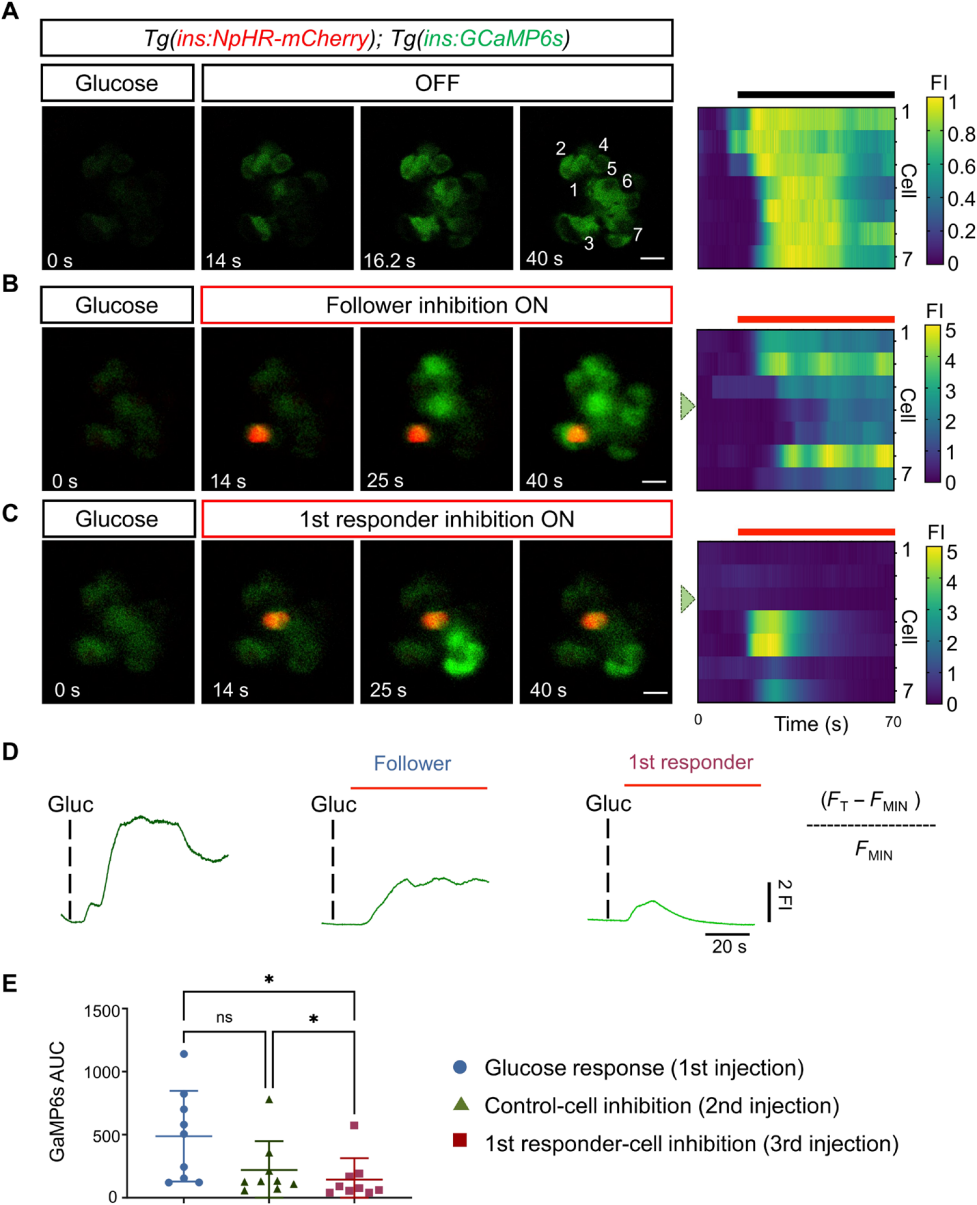
Optogenetic inhibition of first-responder cells using NpHR3.0 decreases the pan-islet glucose-stimulated calcium influx. (**A**) Images and raster plots from a time-lapse recording (6 Hz) of the islet after a glucose stimulation in *Tg(ins:GCaMP6s); Tg(ins:eNpHR3.0-mCherry)* double transgenic larvae. (**B** and **C**) Images from the islet shown in (A) after a glucose injection and simultaneous in vivo optogenetic inhibition with a green laser (λ = 561). The optogenetic inhibition was performed with the region of interest (ROI) scan encompassing the area of one follower or a first-responder β cell. Glucose was injected at 5-min intervals. The red signal corresponds to NpHR-mCherry fluorescence. (**D**) The traces show the normalized GCaMP6 fluorescence traces and the peak in calcium influx after glucose stimulation and upon the light-mediated inhibition of a control or a first-responder β cell. FI, fluorescence intensity. The red bars indicate the period of green laser exposure. (**E**) Quantification of the area under the curve (AUC) reflecting 250 frames (37.5 s) of normalized GCaMP6 fluorescence after glucose injection, follower-cell inhibition, and first-responder cell inhibition (*n* = 9 independent samples) [one-way paired analysis of variance (ANOVA), Tukey’s correction. *P = 0.0226 “glucose response versus first-responder cell” and *P = 0.0259 “control cell versus first-responder cell”; ns, not significant). Data are shown as means ± SD. Scale bars, 10 μm.

Having demonstrated the feasibility of NpHR for β cell inhibition in vivo, we dissected the role of individual cells in coordinating Ca^2+^ dynamics across optical sections of the islet. We used *Tg(ins:GCaMP6s); Tg(ins:eNpHR3.0-mCherry)* double transgenic larvae. First, we induced a pulse of glucose to identify the first-responder cell (Fig. 2A). Subsequently, we gave additional pulses of glucose and simultaneously activated NpHR by illumination with the green laser (λ = 561), targeting either the presumptive first responder or a follower cell (Fig. 2, B and C). First-responder inhibition produced the strongest dampening of calcium influx as compared to follower-cell inhibition (Fig. 2, D and E). In the example shown in Fig. 2C, a small group of β cells showed an increase in GCaMP6s fluorescence upon inhibition of the first-responder cell, suggesting that a different cell had initiated the Ca^2+^ response albeit with lower efficiency, leading to the generation of a partial response (Fig. 2C and movies S3 to S5).

### An optogenetic system for in vivo activation of β cells identifies cells with disproportionate control over Ca^2+^ dynamics

We next asked whether first-responder activation by light-regulated depolarization would trigger an influx of Ca^2+^ in the follower cells even in the absence of glucose stimulation. To this end, we generated a transgenic line expressing the blue-light–driven cation pump CheRiff under the control of the zebrafish insulin promoter (*23*). We then combined the CheRiff transgenic line with a red calcium reporter, *Tg(ins:K-GECO1)*. The genetically encoded red calcium indicator K-GECO1 has a similar design to the GCaMP calcium reporter (*27*), and its use here allowed us to image Ca^2+^ simultaneously while controlling membrane potential via optogenetic regulation of CheRiff–green fluorescent protein (GFP). First, we explored whether CheRiff-based optogenetic activation is sufficient to induce Ca^2+^ influx in the islet. We imaged the islets of *Tg(ins:K-GECO1);Tg(ins:CheRiff-GFP)* larvae and then activated the CheRiff by illuminating the entire islet with a blue laser (λ = 470 nm) for 30 s while simultaneously recording Ca^2+^ dynamics. We found that it was possible to trigger an influx of calcium upon pan-islet optogenetic activation in vivo (movie S6).

**Fig. 3.**
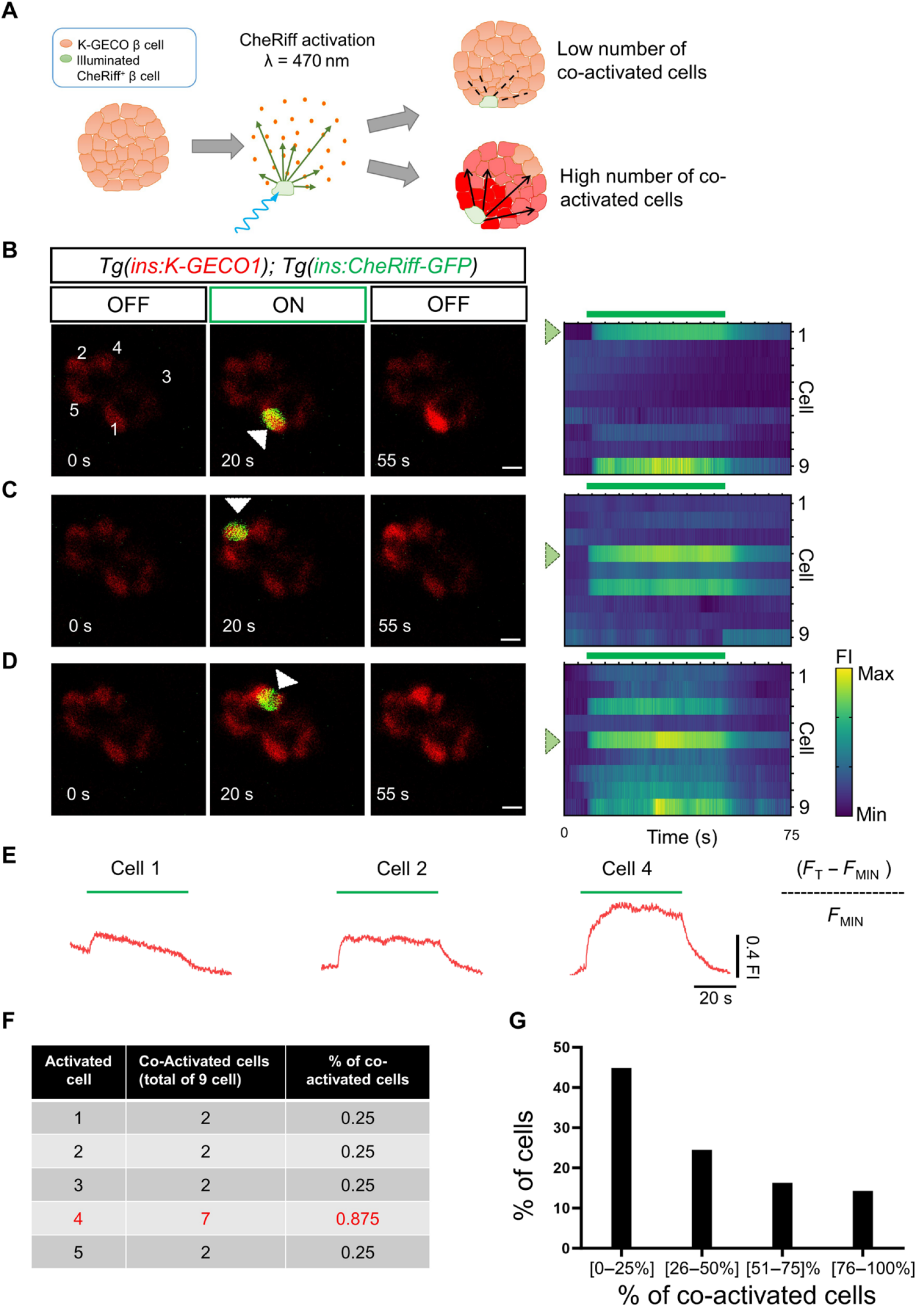
Optogenetic activation using CheRiff reveals the presence of a functional hierarchy of β cells. (**A**) Cartoon representing the rationale for optogenetic interrogation of individual β cells. (**B** to **D**) Images and raster plots from calcium recoding and simultaneous in vivo optogenetic activation with a blue laser (λ = 470). The time-lapse recording was performed at 6 Hz in *Tg(ins:K-GECO1);Tg(ins:CheRiff-GFP)* double transgenics. The green signal corresponds to CheRiff-GFP fluorescence. The optogenetic activation was performed with an ROI scan encompassing the area of one cell. The traces (**E**) show the cumulative normalized K-GECO1 fluorescence for the cells outside the area of laser illumination. The blue bars indicate the period of blue-laser exposure. White arrowheads point to the illuminated cells. Scale bars, 10 μm. (**F**) The table shows the number of β cells co-activated by illuminating individual β cells shown in B. (**G**) Chart showing the functional hierarchy of β cells (n = 49 cells from 10 independent samples). Only ~14% of β cells can propagate the calcium across >75% of the cells in the imaging plane. Data are shown as mean ± SD. Scale bars, 10 μm.

Having established this optogenetic system, we performed single-cell depolarization experiments (Fig. 3, A to E, and movies S7 to S9). We selected the center of the cell using a region of interest (ROI) covering a circle with an approximate diameter of 10 μm, ensuring that a single cell in the plane of illumination is activated. We found that, upon individual activation, ~45% of β cells did not co-activate more than their immediate neighbor β cells. Around 24% of β cells activated four to five additional cells (25 to 75% of the cells). Notably, ~14% of the cells showed high potential for coordination, as they co-activated most of the cells in the imaging plane (>75% of cells; >5 cells) (Fig. 3, F and G) (*n* = 10 islets). Thus, we find evidence for a functional hierarchy among β cells using two different optogenetic systems.

### First-responder cells trigger Ca^2+^ activity in follower cells

**Fig. 4.**
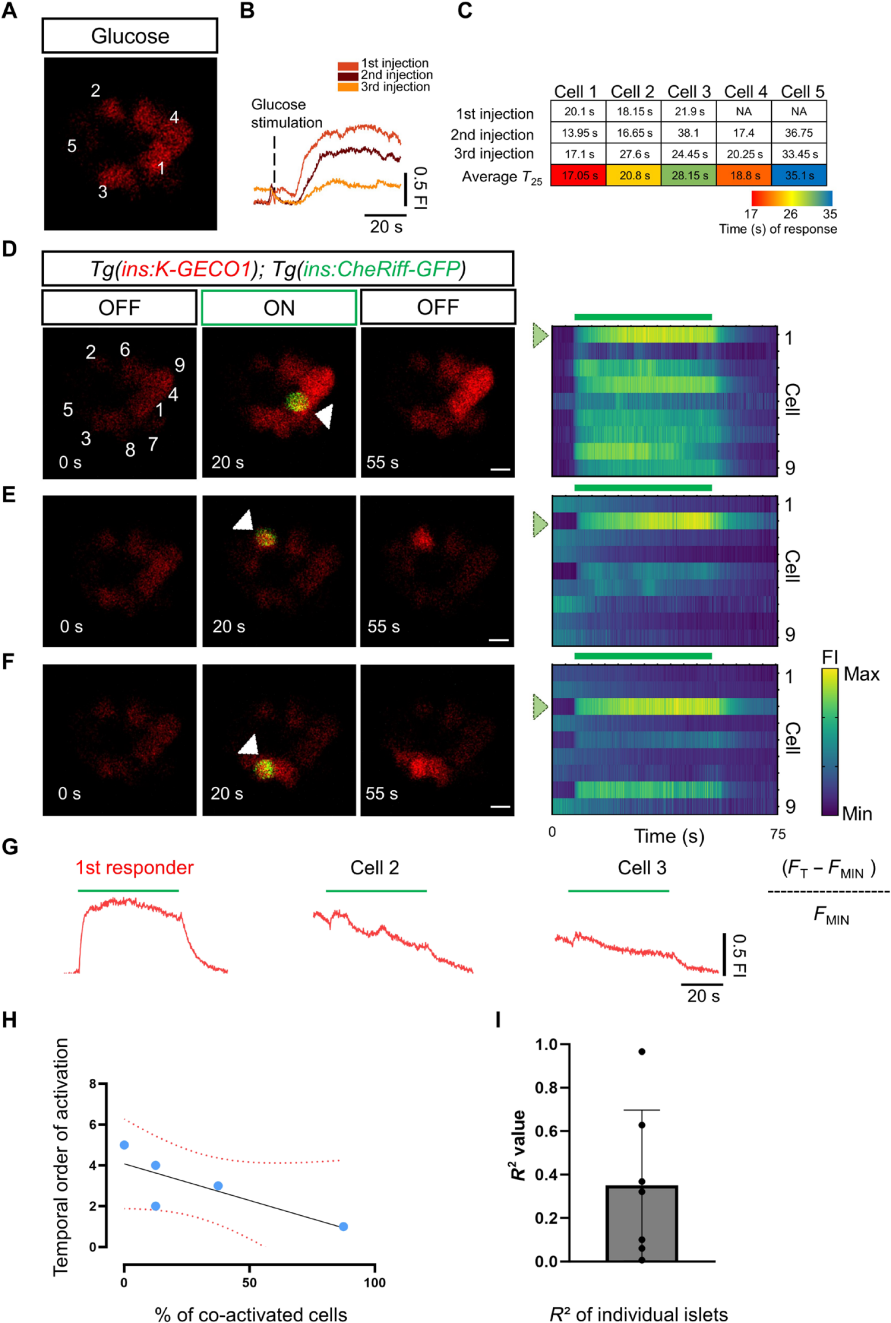
Combining CheRiff activation with glucose stimulation shows that first responders occupy the top of the functional hierarchy. (**A**) Image from a time-lapse recording at 6 Hz of the islet after a glucose injection. The traces (**B**) show the cumulative normalized K-GECO1 fluorescence traces after glucose stimulation. Glucose was injected at 5-min intervals. (**C**) The table shows the time of response for individual cells (K-GECO1 fluorescence increase to >25% above baseline). The cells are numbered according to their position in (A). The table shows a color key of the relative time of response for individual cells to each glucose injection. (**D** to **F**) Representative images and raster plots from the time-lapse recordings show the optogenetic activation of cells from the islet in (A). The optogenetic activation was performed via an ROI scan encompassing the area of one cell. The white arrowhead points to the illuminated β cell. (**G**) The traces show the cumulative normalized K-GECO1 fluorescence traces for β cells outside of the area of illumination. The blue bar indicates the period of blue laser exposure. (**H**) Graph plotting the percentage of co-activated β cells versus the time of response for each cell. The dotted line shows a linear regression and the associated *R*^2^ value. Each dot represents one cell. (**I**) The average *R*^2^ value from seven samples was 0.316 with a ±SD of 0.265 (*n* = 39 cells from seven independent samples). Data are shown as means ± SD. Scale bars, 10 μm.

Our noninvasive technology enabled us to address whether β cells with the highest capability for co-activating cells in the islet coincide with the population of first-responder β cells (*11*). To this end, we first gave three pulses of glucose while recording the calcium responses to identify the first-responder cell (Fig. 4, A to C, and movie S10). We then proceeded to interrogate individual β cells via CheRiff optogenetics in the same islet (Fig. 4, D to F, and movies S11 and S12). We found that, in five of the seven larvae, the first-responder cell was that with the highest potential of recruitment of other β cells, as its activation triggered co-activation of most of the cells in the imaging plane (39 cells from seven different animals) (Fig. 4, G and H). In general, the analysis of multiple islets revealed a weak but positive correlation between the temporal order of response and the ability of the cell to co-activate other cells i.e., the cells that tend to respond faster can co-activate more cells (Fig. 4I) (*R*^2^ = 0.35 and ± SD 0.34). Consistent with our previous optogenetic experiment (Fig. 3), only 11% of β cells (4 of the 39 cells) triggered co-activation of >75% of the cells in the imaging plane (fig. S2) (*n* = 7 islets). These results demonstrate the stronger capability of first-responder cells compared to follower cells to trigger Ca^2+^ activity in the islet.

### Photoconvertible tracking of β cells reveals the stability of first-responder cells

The above experiments revealed the role of first-responder cells in initiating Ca^2+^ responses. We next sought to explore the molecular features of these cells, which may contribute to their ability to serve in this role. As a first step in achieving this goal, we determined whether first responders are stable over time. To this end, we generated a reporter to track individual β cells stably by single-cell labeling using photo-conversion. This transgenic line expressed a green-to-red photo-convertible mEos2 protein fused to histone 2B (H2B) (*28*) specifically in β cells. Using confocal microscopy, we successfully illuminated individual nuclei with a short pulse of violet light (405 nm), leading to selective green-to-red photo-conversion of mEOS in the cell of choice (fig. S3A). Using the double transgenic line *Tg(ins:K-GECO1);Tg(ins:H2B-mEOS2b)*, we applied three pulses of glucose and recorded Ca^2+^ responses (fig. S3, B and C). We identified in situ the first-responder cell and specifically photo-converted its nucleus (fig. S3, D and E). Twenty-four hours after the photo-conversion, we repeated the glucose stimulation (fig. S3, F and G, and movie S13 to S15). We found that, in 80% of the cases (four of the five cells, *n* = 5 larvae), the photolabeled cell was again a first responder (fig. S3, H and I). This suggests that first-responder cells represent a population stable for at least 24 hours, which agrees with recent results from cultured mouse islets (*12*).

### The speed of glucose uptake does not define the first-responder cell

The ability of first-responder cells to initiate the Ca^2+^ response may depend on a more rapid uptake of glucose compared to follower cells. To test this hypothesis, we used the red calcium reporter *Tg(ins:K-GECO1)* in combination with the green glucose analog 2-NBDG [(2-(7-nitrobenz-2-oxa-1,3-diazol-4-yl)amino)], which reports glucose uptake into individual cells (*29*). We first recorded the calcium response to unlabeled glucose to identify the first responder followed by an injection of 2-NBDG to monitor the glucose uptake (fig. S4, A to H). We ranked individual cells according to the relative time of increase in both Ca^2+^ and 2-NBDG (fig. S4, I and J). Plotting the time of Ca^2+^ increase versus glucose uptake revealed no correlation (*R*^2^ = 0.065) (fig. S4, K and L). These results suggest that differences in glucose uptake alone do not explain the pattern of glucose response observed upon glucose stimulation in vivo.

### Molecular profiling of β cell subpopulations with different sensitivities to glucose

**Fig. 5.**
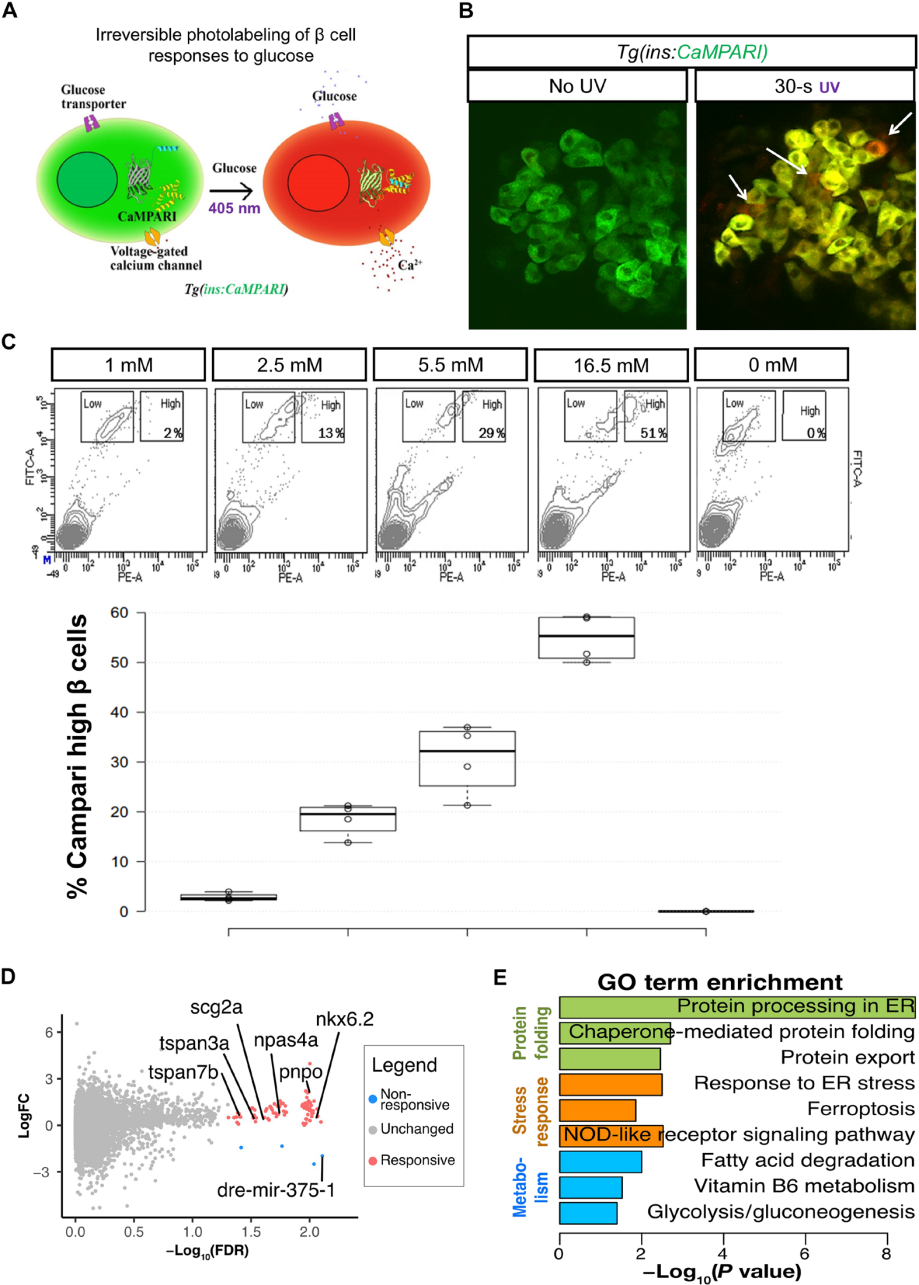
Transcriptional profile of highly glucose-responsive β cells. (**A**) Schematic of CaMPARI-based labeling of glucose sensitive β cells. The *Tg(ins:CaMPARI)* line expresses CaMPARI specifically in the zebrafish β cells. Cells that respond to glucose stimulation have an influx of calcium ions. An increase in intracellular calcium, along with exposure to ultraviolet (UV) light irreversibly converts the fluorescence of CaMPARI from green to red. (**B**) Ex vivo labeling of glucose responsiveness. Confocal image of the primary islets from 30-dpf (days postfertilization) *Tg(ins:CaMPARI)* animals incubated in 2.5 mM glucose and not exposed to UV (left) or with 30 s of UV exposure (right). UV exposure induces green-to-red photoconversion (arrows). (**C**) Representative fluorescence-activated cell sorting (FACS) profiles (top) and quantification (bottom) of *Tg(ins:CaMPARI)* islets exposed to varying amounts of glucose and UV ex vivo. The *x* axis represents red fluorescence [phycoerythrin (PE)], while the *y* axis represents green fluorescence [fluorescein isothiocyanate (FITC)]. Square boxes outline the cells with a low red-to-green ratio (nonresponsive) and with a high red-to-green ratio (responsive). The percentage in the high box represents the proportion of responsive β cells. The quantification represents the percentage of responsive β cells as a boxplot (*n* = 4 independent samples per condition). (**D**) Volcano plot representing differentially expressed [false discovery rate (FDR) < 0.05] between β cells responsive at 2.5 mM and β cells unresponsive at 16.5 mM. Genes enriched in responsive cells are labeled in pink, while genes enriched in nonresponsive cells are labeled in blue. LogFC, log fold change. (**E**) Gene ontology (GO) analysis for differentially expressed genes. Data shown as means ± SD. ER, endoplasmic reticulum. NOD, nonobese diabetic.

Because all of the cells interrogated above appeared to show uniform uptake of glucose, i.e., the speed of glucose uptake does not define the first-responder cell, we next explored whether first-responder β cells may differ from followers in their intrinsic sensitivity to glucose involving subsequent metabolic steps. Classical studies in rodent islets have documented that β cells show a range of glucose sensitivities, with 5% of β cells responding to glucose levels as low as 1 mM, whereas the remaining 95% are inactive [reviewed in (*30*)]. To define the gene expression of highly glucose-sensitive cells, we used the genetically encoded calcium indicators, called CaMPARI (*31*) that enables one to permanently mark cells that have experienced an increase in intracellular Ca^2+^ levels. We thus generated a transgenic line with β cell–specific expression of CaMPARI (Fig. 5A). When islets from *Tg(ins:CaMPARI)* fish are exposed to glucose and violet light, CaMPARI fluorescence permanently converts from green to red in those β cells that experienced an increase in glucose-stimulated intracellular calcium (Fig. 5B). Conversely, β cells that do not respond to glucose during the period of violet-light exposure maintain green fluorescence, allowing the responsive and nonresponsive β cell subpopulations to be distinguished. When zebrafish islets were treated with increasing levels of glucose ex vivo, there was a proportional increase in β cells that had undergone photoconversion (Fig. 5C), validating the suitability of the method to label β cells with different thresholds of glucose-stimulated Ca^2+^ influx.

Using CaMPARI and fluorescence-activated cell sorting (FACS), we separated individual β cells from adult islets according to their fluorescence levels. We focused on the two extremes of the glucose-responsiveness spectrum: those β cells that responded to low (2.5 mM) glucose (highly responsive) and those that did not respond even to very high (16.5 mM) glucose (nonresponsive). The two populations of β cells were transcriptionally profiled at the single-cell level. Comparison between β cells (*n* = 92 cells each from three animals) with low and high glucose responsiveness identified 81 differentially expressed genes (Fig. 5D), of which 77 were up-regulated in responsive β cells and four genes were up-regulated in nonresponsive β cells (data S1). Unbiased gene ontology (GO) analysis revealed that the differentially regulated genes were involved in protein folding processes such as “protein processing in endoplasmic reticulum,” “chaperone-mediated protein folding,” “protein export,” as well as in stress response processes such as “response to ER stress,” “ferroptosis,” and “NOD-like receptor signaling” (Fig. 5E). In addition, genes involved in metabolism (“fatty acid degradation,” “vitamin B6 metabolism,” and “glycolysis/gluconeogenesis”) were identified to be differentially regulated. Within these pathways, the responsive β cells displayed enrichment of the activity-induced transcription factor *npas4a* (*32*); *scg2a*, an insulin granule–associated gene implicated in β cell function (*33*, *34*); as well as *tspan7b*, a transmembrane protein with roles in insulin secretion (*35*) whose human ortholog represents one of the auto-antigens associated with T1D (*36*). The unresponsive cells showed elevated Mir-375 and *txnipa* expression, both of which act as negative regulators of insulin production in mammalian cells (Fig. 5D) (*37*, *38*). These results suggest a distinct functional, metabolically active, and vulnerable state in highly glucose-responsive β cells.

### First-responder β cells show enrichment for the vitamin B6 synthesis enzyme *pnpo*

We further explored the possibility that some of the genes associated with the highly glucose-responsive β cells demarcate the first-responder cell population as well. We focused our analysis on *pnpo*, encoding a rate-limiting enzyme for the production of pyridoxal phosphate (PLP), the active form of vitamin B6. PLP is an essential cofactor for the synthesis of neurotransmitters [γ-aminobutyric acid (GABA), serotonin, and dopamine] and for the functioning of mitochondrial pathways involved in amino acid biosynthesis, energy metabolism, and metal homeostasis (*39*). Moreover, *pnpo* (*sgll*) activity in insulin-expressing neurons has been associated with glucose regulation in *Drosophila* (*40*), and there are strong epidemiological links between vitamin B6 deficiency and metabolic disease (*41*), making *pnpo* a relevant candidate to explore.

We first analyzed *pnpo* expression in our previously generated datasets of single-cell RNA expression of zebrafish pancreas. Notably, these data show that *pnpo* is expressed detectably in a small population of β cells (4% of insulin-expressing cells) (fig. S5, A to D). This group of cells appears to experience heightened metabolic performance. “Cellular respiration, mitochondrial ATP synthesis coupled electron transport,” “respiratory electron transport chain,” “protein peptidyl-prolyl isomerization,” “aerobic respiration,” and “intra-Golgi vesicle-mediated transport” are among the top pathways associated with *pnpo*^hi^ cells, according to GO analysis (fig. S5, E and F). A large number of genes involved in mitochondrial function, assembly, and adenosine 5′-triphosphate (ATP) production were up-regulated in *pnpo*^hi^ cells (fig. S5E and data S2) (*uqcc3*, *cox6a1*, *atp5e*, *nduf7*, *atpif1b*, *cox5b*, *cox8a*, *atp5c1*, and *sdha*). In addition, there is up-regulation of genes involved in vesicle secretion and protein folding including *calm2b*, *cope*, *arf5*, *ywhaqa*, *wdr83os*, *fkbp5*, *fkbp1aa*, *tcp1*, *copz2*, *atf6*, and *scg3*.

To assess the expression of *PNPO* mRNA in human islets, we analyzed publicly available datasets of single-cell transcriptomics of pancreas donors provided by the Human Pancreas Analysis Program (HPAP Consortium). Consistent with our findings in zebrafish, a small proportion of β cells were positive for *PNPO* in the human pancreas (11%) (fig. S6, A to D). Compared to non-expressing cells, the *PNPO*^+^ cells were characterized by the enrichment for genes involved in “insulin secretion,” “carbohydrate digestion and absorption,” “AMPK signaling,” “maturity onset diabetes of the young,” “type I diabetes mellitus,” “type II diabetes mellitus,” “Parkinson disease”, etc., according to the Kyoto Encyclopedia of Genes and Genomes (KEGG) (fig. S6, E and F, and data S2).

**Fig. 6.**
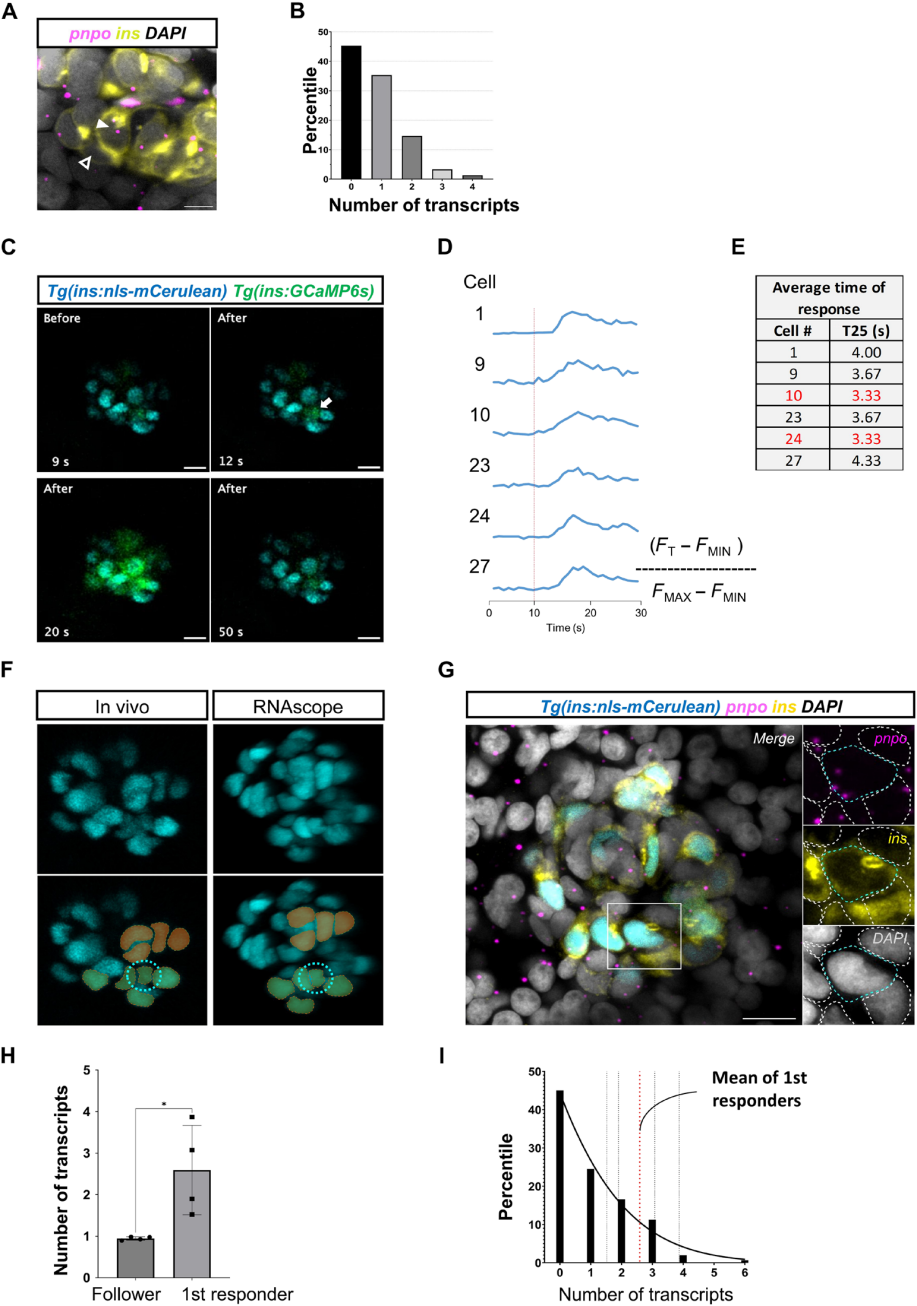
*pnpo* marks first-responder β cells. (**A**) RNAscope analysis of *pnpo* expression in β cells in the islets of zebrafish larvae (5 dpf). (**B**) Individual β cells show a heterogeneous number of *pnpo* transcripts. Most cells have zero transcripts. A small proportion (5%) of the cells have three and four transcripts (*n* = 5 independent samples). β Cells are shown in yellow using insulin immunofluorescence and *pnpo* transcripts are in magenta. The white arrowhead points to a *pnpo*-positive β cell with two *pnpo* transcripts. The dark arrowhead points to a *pnpo-*negative β cells. (**C**) Representative Ca^2+^ responses to glucose injection. The images represent maximum projections of confocal slices spanning the whole islet. (**D**) Fluorescent traces from individual selected cells. (**E**) Speed of response of selected cells from the time-lapse imaging. (**F**) The β cells from the in vivo imaging were matched with maps of *pnpo* transcripts following RNAscope analysis of *pnpo* expression. A nuclear fluorescent reporter (blue) was used as for cell matching of β cells and recognizable landmarks (pseudocolors) were used to match the cells. (**G**) The expression of *pnpo* is shown for the first-responder cell identified using the time-lapse shown in (C). β Cells are shown in yellow using insulin immunofluorescence, and *pnpo* transcripts are in magenta. (**H**) Comparison of average number of transcripts between first-responder and follower cells (*n* = 4 independent samples). (**I**) Percentile distribution of β cells according to number of *pnpo* transcripts (*n* = 4 independent samples). The mean transcript number for first-responder β cells is indicated with a red line. Unpaired two-tailed *t* tests; *P = 0.0286. Data are shown as means ± SEM. Scale bars, 10 μm.

To validate the presence of β cells with distinct levels of *pnpo* expression in situ, we applied RNAscope technology, which offers the capability for single-molecule RNA detection. This method showed that 45% of β cells had no detectable *pnpo* expression, 35% had only one *pnpo* transcript, 15% had two transcripts, and only 5% of the cells showed three and four transcripts (Fig. 6, A and B). These results indicate that β cells are highly heterogeneous in terms of *pnpo* expression levels with only a small group of cells being *pnpo*^hi^, consistent with our single-cell RNA transcriptomic data.

The hierarchical enrichment of *pnpo* in a small subset of β cells reminded us of the first responder and follower organization of the cells. Therefore, we hypothesized that *pnpo* marks the first-responder cell population. To test this possibility, we first recorded the Ca^2+^ response to glucose stimulation in three-dimensional (3D) videos, and, subsequently, we fixed the samples and monitored the expression of *pnpo* in each β cell (Fig. 6, C to E, and movie S16). For these experiments, we used nuclear labeling with a blue fluorescent protein, which, in contrast to other fluorophores that we tested, such as mCherry and mEOS, was able to withstand bleaching during RNAscope implementation. Through 3D correlative image analysis, we identified the first responder in vivo and in situ and overlayed this information with the maps of *pnpo* transcript distribution in each β cell (Fig. 6, F and G). This analysis revealed that first responders were enriched for *pnpo* transcripts compared to follower cells (Fig. 6H) and ranked in the top 16 percentile of all β cells in terms of expression levels (Fig. 6I). These data provide support for the notion that there is an overlap between the first-responder and *pnpo*^hi^ β cells.

### Vitamin B6 deficiency leads to impaired β cell Ca^2+^ response to glucose

**Fig. 7.**
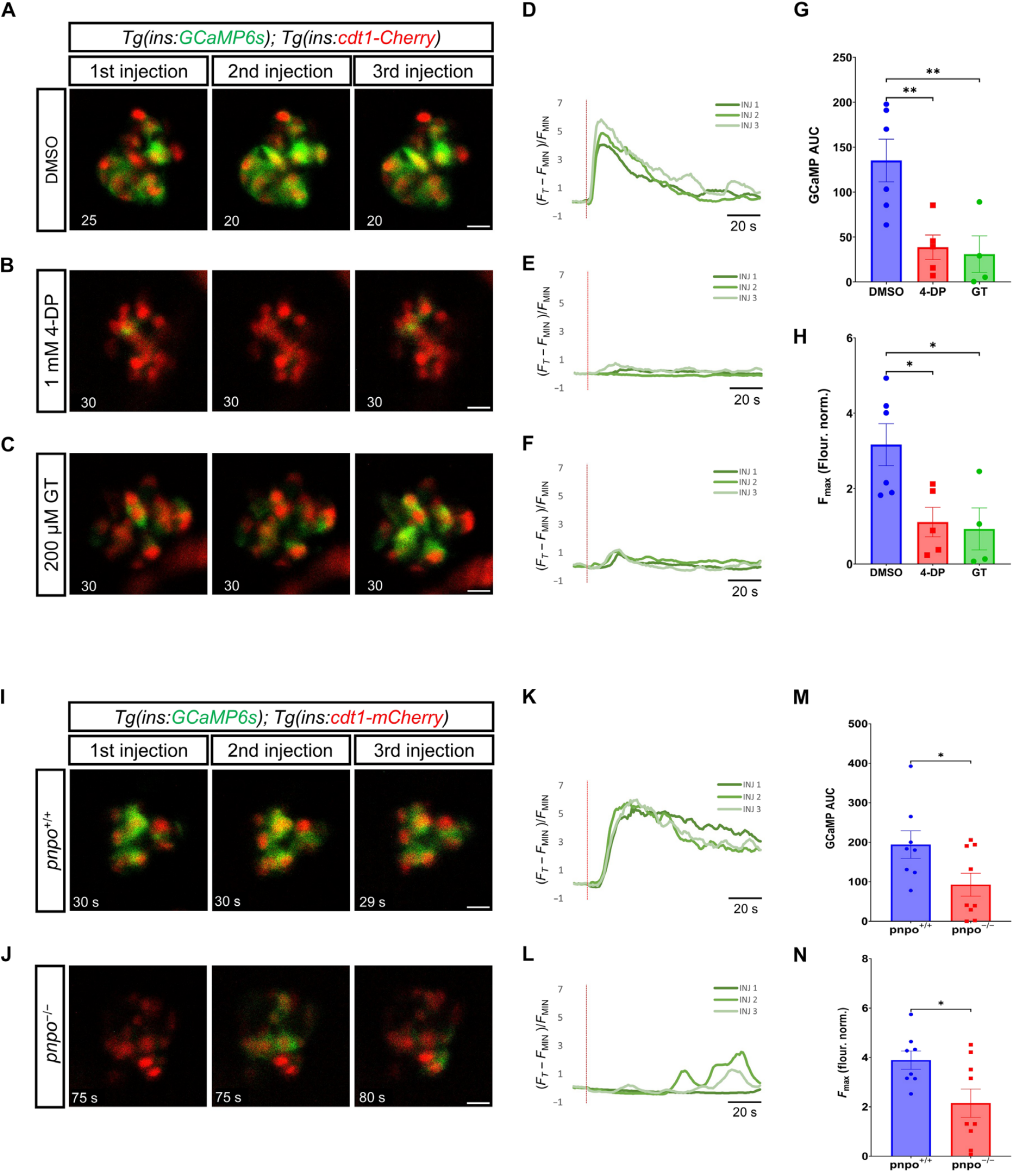
*pnpo* and vitamin B6 are necessary for the β cell Ca^2+^ response to glucose in zebrafish. (**A** to **C**) Time lapse of β cell Ca^2+^ activity upon triple-glucose stimulation of *Tg(ins:GCaMP6s);Tg(ins:cdt1-mCherry)* larvae treated with dimethyl sulfoxide (DMSO), 1 mM 4-deoxypyridoxine (4-DP), or 200 μM ginkgotoxin (GT). The panels show frames with high β cell activity following each stimulus (single focal-plane imaging at 1 Hz for 120 s). Glucose injection was performed at the 10th second of each recording. The time stamp shows time relative to the start of recording. (**D** to **F**) Normalized GCaMP fluorescence traces. Red lines indicate glucose injection. (**G**) AUC of normalized GCaMP fluorescence after glucose injection for DMSO, 4-DP, and GT. Each dot represents the average of three glucose stimulations per sample (*n* = at least four independent samples per group). One-way ANOVA with Dunnett’s correction. **P = 0.0090 for DMSO versus 4-DP; **P = 0.0084 for DMSO versus GT. (**H**) Maximum GCaMP fluorescence (*F*_max_) recorded throughout the imaging for DMSO, 4-DP, and GT (*n* = at least four independent samples per group). One-way ANOVA with Dunnett’s correction. *P = 0.0235 for DMSO versus 4-DP; *P = 0.0210 for DMSO versus GT. (**I** and **J**) Time lapse of β cell Ca^2+^ activity in *pnpo*^+/+^ and *pnpo*^−/−^ larvae (5 dpf). Imaging parameters as in (A). (**K** and **L**) Normalized fluorescence traces. Red lines indicate glucose injection. (**M**) AUC of normalized GCaMP fluorescence for *pnpo*^+/+^ and *pnpo*^−/−^. Each dot represents the average of three glucose stimulations per sample (*n* = at least eight independent samples per group). Unpaired two-tailed *t* test, *P = 0.0388. (**N**) Maximum GCaMP fluorescence (*F*_max_) recorded throughout the imaging for *pnpo*^+/+^ and *pnpo*^−/−^. Unpaired two-tailed *t* test, *P = 0.0248. Each dot represents the average of three stimulations (*n* = at least eight independent samples per group). Data are shown as means ± SEM.

**Fig. 8.**
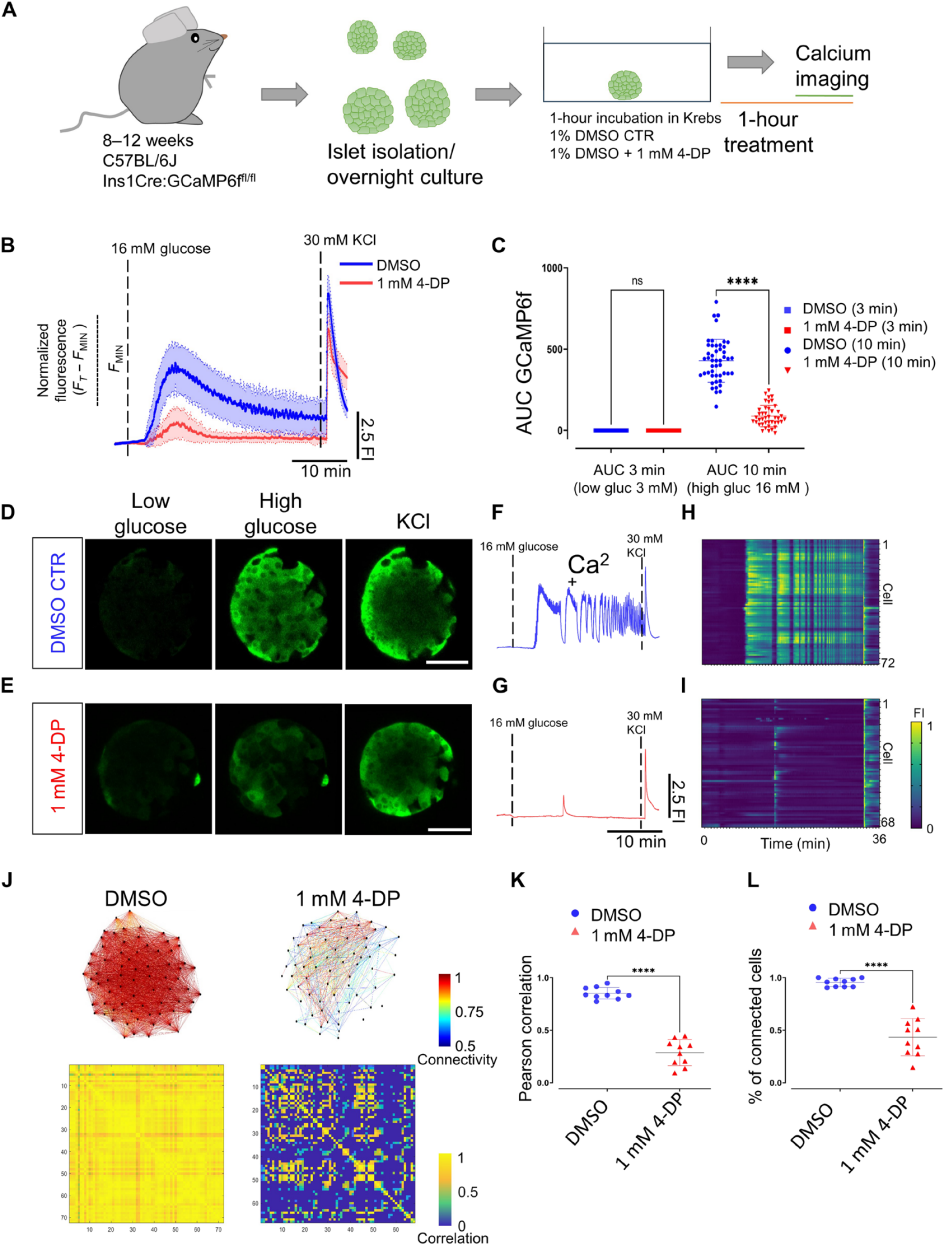
Vitamin B6 antagonism reduces the Ca^2+^ response and the β cell coordination in mouse islets. (**A**) Experimental design for in vitro calcium imaging of islets from male C57BL/6J Ins1Cre:GCaMP6f^fl/fl^ mice aged 10 weeks with 4-DP treatment. (**B**) Average calcium fluorescent traces from control and 1 mM 4-DP–treated islets. The blue and red lines show average GCaMP6f fluorescent traces and SDs from the imaged islets. (**C**) AUC quantifications from controls and 1 mM 4-DP–treated islets at 3 and 10 min. Each dot represents an islet (one-way paired ANOVA, Tukey’s correction ****P = 1.60 × 10^−73^; 48 islets for 1% DMSO controls and 42 islets for 1 mM 4-DP, from three mice). (**D** and **E**) Snapshots from a time-lapse recording of the individual islet at 0.5 Hz before and after glucose stimulation and KCl depolarization from control and 1 mM 4-DP treatment. (**F** and **G**) Normalized GCaMP6f fluorescence traces upon glucose stimulation (16 mM glucose) and 30 mM KCl depolarization from control and 1 mM 4-DP–treated islet shown in (D) and (E). (**H** and **I**) Raster plots show the GCaMP6f signal for individual cells from islets shown in (D) and (E). (**J**) Connectivity map and Pearson correlation matrix of the islets in (D) and (E) during the first-phase response. (**K**) Pearson correlation of individual islets (unpaired two-tailed Student’s *t* test. ****P = 3.87 × 10^−8^) during the first-phase response. (**L**) Percentage of connected cells of individual islets during the first-phase response. *n* = 10 islets from three mice (1% DMSO) and *n* = 10 islets from three mice (1 mM 4-DP) (unpaired two-tailed Student’s *t* test. ****P = 9.47 × 10^−11^). Data are shown as means ± SD. Scale bars, 40 μm.

To evaluate the involvement of vitamin B6 in the β cell Ca^2+^ activity, we used small-molecule inhibitors of the pathway. 4-Deoxypyridoxine (4-DP) can interfere with Pnpo and the pyridoxal kinase Pdxk, whereas Ginkgotoxin (GT) acts on pyridoxal kinase Pdxk (*42*–*44*). The two enzymes cooperate in the production of the biologically active form of vitamin B6. We treated *Tg(ins:GCaMP6s); Tg(ins:cdt1-mCherry)* double transgenic larvae with either 1 mM 4-DP or 200 μM GT for 2 hours and performed Ca^2+^ imaging with simultaneous glucose stimulation. Both the 4-DP and the GK treatments led to an impaired Ca^2+^ response in β cells, with only a few cells mildly responding to the glucose stimulation, indicating a lack of coordinated Ca^2+^ influx among cells (Fig. 7, A to F, and movies S17 to S19). Compared to dimethyl sulfoxide (DMSO)–treated controls, the overall Ca^2+^ response of β cells in 4-DP and GT groups was significantly lower, as shown by both the area under the curve (AUC) and the maximum fluorescence intensity (*F*_max)_ values (Fig. 7, G and H). Moreover, we used a recently established *pnpo* knockout zebrafish line to gather further evidence for our hypothesis (*45*). We performed Ca^2+^ imaging in the wild-type (WT; *pnpo*^+/+^) and *pnpo* knockout (*pnpo*^−/−^) siblings in the *Tg(ins:GCaMP6s); Tg(ins:cdt1-mCherry)* double transgenic background. Most *pnpo* knockout animals could not properly respond to the glucose stimulus in three injections, some showing mild responses in a few cells in different injections (Fig. 7, I to L). This is while the WT group imaged in parallel showed normal Ca^2+^ responses to the three stimulations (movies S20 and S21). The whole-islet AUC and *F*_max_ values were lower in the *pnpo*^−/−^ group (Fig. 7, M and N). To determine whether vitamin B6 has a regulatory role in mouse calcium dynamics, we applied 4-DP to cultured mouse islets and measured their Ca^2+^ response to glucose. Mouse islets treated with 1 mM 4-DP also showed a significant reduction in calcium activity in response to glucose as reflected by the AUC quantifications (Fig. 8, A to I, and movies S22 and S23). We found a dose-response relationship between 4-DP treatment and Ca^2+^ dynamics. While 0.5 mM 4-DP treatment did not show a significant decrease in AUC measurements, 2 mM 4-DP treatment for 1 hour completely abolished calcium responses in mouse islets (figs. S7 and S8). 4-DP–treated islets (1 mM) showed a delay of 2.1 min in the onset of the first-phase response (average *T*_25_ of 5.6 min ± 1.15) in comparison to DMSO-treated controls, which presented an average *T*_25_ of 3.4 min ± 0.75 (fig. S9, A to C). Similarly, peak amplitude and peak width during the first and second phases exhibited a dose-dependent reduction (fig. S9, D to G). Connectivity analysis during the first-phase response showed that the Pearson *R*–based correlation dropped from 0.85 ± 0.05 to 0.28 ± 0.12 in 1 mM 4-DP–treated islets (Fig. 8, J to L, and fig. S10). Likewise, the average number of connected cells during the first phase-response (defined as those with a positive Pearson *R*–base correlation of >0.2) was significantly reduced from an average of 95 ± 3% in controls to 40 ± 17% in 1 mM 4-DP–treated islets (Fig. 8, J to L, and fig. S10). Similar results were obtained during the second phase-response (fig. S10). These experiments show that vitamin B6 is required for mouse islets to efficiently connect the functional cellular network in response to glucose. Thus, vitamin B6 production not only is a marker of the first-responder cells but also is involved in the proper functioning of β cells.

## DISCUSSION

Here, we describe complementary intravital approaches to optically interrogate β cells in vivo in the living zebrafish larva. Thus, we show that the light-gated Cl^−^ ion pump halorhodopsin (NpHR) can be applied to inhibit β cell depolarization in vivo. We also implemented the optically orthogonal system of the red calcium indicator K-GECO1 in combination with the blue-shifted CheRiff to depolarize individual β cells. By interrogating functional properties cell by cell, we show the existence a functional heterogeneity that underlies the coordination during the first-phase response to glucose stimulation. The hierarchy encompasses cells with different levels of influence over the islet, including first responder and followers. Notably, we demonstrate that the islet includes a first-responder β cell subpopulation with distinct functional, metabolic, and molecular characteristics versus “follower” cells. We note that highly connected hub cells were not explored in this system given the absence of sustained (second phase) Ca^2+^ oscillations.

**Fig. 9.**
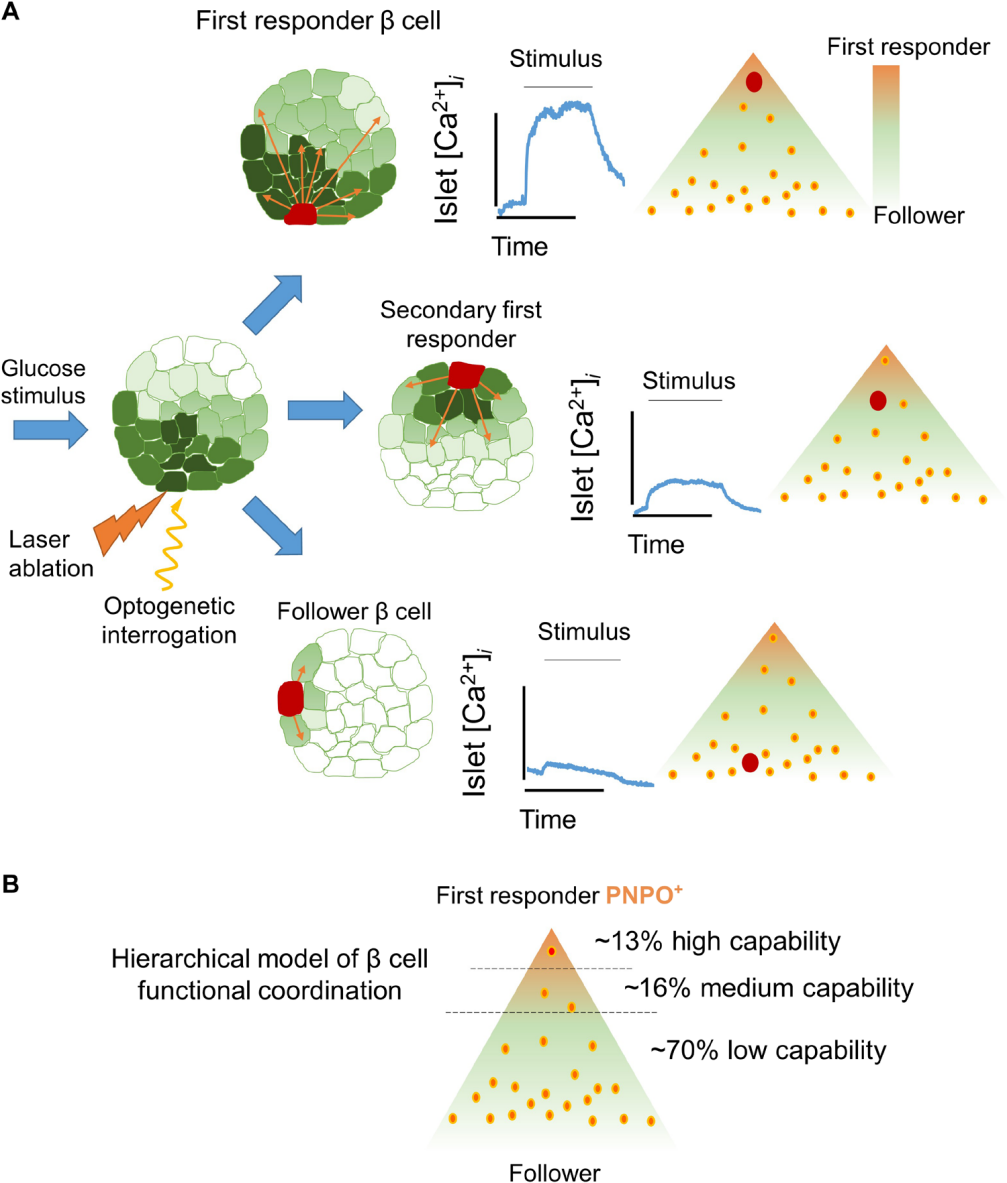
Model of coordination based on β cell hierarchies. (**A**) The functional characterization of islet coordination via optogenetics leads us to propose a hierarchical model for β cell coordination. The coordination involves first-responder cells at the top of the hierarchy, as well as follower cells at the bottom. (**B**) First-responder β cells are characterized by higher expression of the vitamin B6 production enzyme *pnpo*. The first-responder β cells are capable of triggering a Ca^2+^ response across many cells. Some β cells present medium to low capability to co-activate other β cells. The rest of the β cells are followers because they present a low capability to recruit β cells. The co-activation capability of the cells and their proportions were determined on the basis of the pooled data from the CheRiff optogenetic analysis.

Together, our results lead us to propose a hierarchical model of islet coordination in the zebrafish (Fig. 9). In contrast to the majority of β cells, first-responder cells occupy the top levels of the hierarchy, being capable of coordinating the majority of the islet’s β cells. These cells are also highly glucose-responsive, as they show an influx of calcium earlier than most of the cells upon glucose stimulation. We also identify *pnpo* as a molecular marker of first-responder β cells, and, through pathway analysis, we find that, across species, first-responder cells are characterized by a distinct metabolic and molecular state compared to the rest of β cells. Last, we show that a heterogeneous expression of the vitamin B6 production enzyme *pnpo* is essential for the Ca^2+^ response to sugar.

In a previous study (*9*), we showed that electrical silencing via halorhodopsin (NpHR) of highly connected β cells (hubs) was sufficient to impair Ca^2+^ coordination across individual imaging planes within isolated mouse islets. The identified hub cells were characterized by high levels of glucokinase; two- to fivefold lower expression of insulin, Pdx1, and Nkx-6.1; reduced expression of the sarcoplasmic reticulum calcium/adenosine triphosphatase sarcoplasmic/endoplasmic reticulum Ca2+-ATPase 2 (SERCA2); and hyperpolarization of mitochondria in comparison to other β cells (*9*). Along similar lines, Westacott *et al.*, using the optogenetic activator ChR2, reported that stimulation of ~5% of β cells triggered substantial activation of the islet outside the area used to activate the targeted single cells. The latter authors suggested that areas of β cells with the highest metabolic oscillation frequency, monitored via nicotinamide adenine dinucleotide phosphate [NAD(P)H] fluorescence, may play a major role in governing the frequencies initiating Ca^2+^ wave propagation (*6*). Our present results provide further evidence that the hierarchy of β cells is not “black and white” as also inferred from studies of Ca^2+^ dynamics in mouse islets engrafted into the anterior eye chamber (*11*, *46*) but encompasses cells with different levels of control over the islet. The identified first-responder cells here are characterized by up-regulation of genes involved in mitochondrial respiration, ATP synthesis, protein folding, vesicle trafficking, and secretion. However, it must be emphasized that, because different mechanistic processes may be at work during the first and the second phases of the glucose response, the characteristics of the cells coordinating each of them are likely to differ.

By adopting the photoconvertible Ca^2+^ reporter–CaMPARI from the neuroscience field, we provide a clear “snapshot” of a second layer of functional β cell heterogeneity identifying a highly glucose sensitive β cell subpopulation and likely corresponding to first-responder cells. Using CaMPARI, we transcriptionally profiled β cells that responded at low levels of glucose (2.5 mM), as well as β cells that remained nonresponsive at high levels of glucose (16.5 mM). By comparison of these extremes, we identified 81 genes that differentiate between the two subpopulations. We note that the activation of CaMPARI itself, which involves a rapid photoactivation, is unlikely in itself to impact the gene expression profile significantly over the time course of these experiments.

Mir-375, enriched in nonresponsive β cells, is a negative regulator of insulin secretion and is implicated in β cell proliferation and response to metabolic stress (*38*). Responsive β cells, on the other hand, displayed enrichment in *scg2a*, an insulin granule–associated gene implicated in β cell function (*33*, *34*); *nkx6.2*, a transcription involved in β cell specification (*47*); and *tspan7b*, a transmembrane protein whose human ortholog represents one of the auto-antigens in T1D (*35*,*36*). The existence of these molecular differences is consistent with the stability of discrete populations, arguing against the notion that these simply reflect different, and potentially transient functional “states.” Of interest, molecular profiling of β cells based on the characterization of their electrical properties using Patch-sequencing (Patch-seq) has identified a positive correlation between exocytosis and *NKX6-1*, *TSPAN1*, *TSPAN2*, and *SCG2*, genes belonging to the same family as the ones identified in our screen (*48*).

To identify a molecular marker of first-responder β cells, we focused in more detail on *pnpo*, encoding a rate-limiting enzyme for the conversion of vitamin B6 to PLP, which is needed as a cofactor for the synthesis of neurotransmitters (GABA, serotonin, and dopamine) as well as for the function of numerous metabolic enzymes in the cell (*39*). By combining functional Ca^2+^ imaging with single-molecule RNA detection, we validated *pnpo* as a marker of the first-responder population. Homozygous mutations in *PNPO* in humans often cause dysregulation in metabolism (*49*). Furthermore, genetic interference with the *Drosophila* homolog of *pnpo* (*sgll*) leads to defects in glucose homeostasis maintenance (*40*), and pregnant mice fed with a vitamin B6 deficient diet exhibit glucose intolerance (*50*). Consistent with our results in zebrafish, a pyridoxine-free diet supplemented with 4-DP caused a blunted insulin release in the pancreas of rats (*51*). In light of the ongoing debate, about whether vitamin B6 reduction is a cause or a consequence of diabetes (*52*), our data show that the former is likely true. Future studies on the role of the subpopulation of *pnpo^+^* β cells across species may shed insights into the mechanisms of β cell coordination by first-responder cells, either through metabolic cross-talk, via neurotransmitter production, or by a yet-to-be-identified pathway. Whether and to what extent *pnpo* cells align with the recently defined subsets of mouse β cells enriched for CD81 (*53*), CD24 (*54*), or CD63 (*55*), remains to be explored.

It is worth noting that there is little overlap of first-responder gene expression in this study with the recently published transcriptomic analysis of mouse leader β cells (*10*). This is expected because the two cell populations are likely to be partly distinct (*10*). Specifically, first responders are crucial to initiate the Ca^2+^ response during the initial response to sugar, while leader cells act as wave-initiator cells during subsequent Ca^2+^ peaks under sustained glucose stimulation (*11*, *12*).

In summary, our data provide a framework and insights into the division of labor within the islet in vivo, reinforcing the importance of β cell heterogeneity for islet function and insulin secretion. The paradigm that we develop here, examining the roles of individual cells in coordinating Ca^2+^ dynamics within the functioning islet, as well as correlating glucose-sensitive to transcriptional differences, may apply to other tissues where cell coordination is crucial for proper function. Further studies are required to determine whether first-responder β cells are subject to enhanced metabolic stress and undergo preferential dysfunction or loss in pathogenic conditions such as T1D and T2D.

### Limitations of the study

Our analysis is focused on the zebrafish pancreas during the early stages of development, which are optically accessible. While the islet is fully functional at that stage, one limitation of this approach is that the size of the islet is smaller than the average mouse or human islet. Individual first-responder cells have a stronger influence over smaller islets compared to larger islets. Nevertheless, the small size of the islet allows higher precision in the optical mapping experiments. A second limitation is that 2D imaging was used for most movies, but this is necessary for high-speed imaging. Expanding the imaging to all cells of the islet reduces the imaging speed. While we limit the size of optical illumination to a small area, it is difficult to account for the activation of cells above or under the illumination plane, as well as for out-of-focus illumination due to tissue diffraction. Phylogenetic differences in islet function might exist among vertebrates, yet, we have also shown a fascinating evolutionary conservation in terms of signaling control of β cell function. For example, we recently showed conserved regulation of Ca^2+^ influx by WNT4 in both fish and human β cells and a strong overlap of gene expression programs across pancreatic cell types from zebrafish to humans, supporting the high molecular and cellular conservation of islet function across evolution (*56*, *57*).

## METHODS

### Animal husbandry

Zebrafish WT AB, WIK, and Tupfel Long-Fin (TL) were used in all the experiments. Zebrafish were raised in standard conditions at 28°C. The previously established transgenic lines used in this study were *Tg(ins:GCaMP6s;cryaa:mCherry)* (*58*), *Tg(ins:cdt1-mCherry;cryaa:GFP)* (*59*), *Tg(ins:K-GeCO1)*, *Tg(ins:mEOS2b-H2B;cryaa:CFP)*, and *pnpo* (*45*). All experiments were carried out in compliance with European Union and German laws (Tierschutzgesetz) and with the approval of the Technische Universität Dresden and the Landesdirektion Sachsen Ethics Committees (approval nos. AZ 24D-9168,11-1/2013-14, TV38/2015, T12/2016, and T13/2016, TVV50/2017, TVV 45/2018, and TVV33-2019). All experiments were performed with zebrafish larvae that did not exceed the 5-dpf (days postfertilization) stage, as stated in the animal protection law (TierSchVersV §14). According to the EU directive 2010/63/EU, the use of these earlier zebrafish stages reduces the number of experimental animals, in agreement with the principles of the 3Rs (Replacement, Reduction, and Refinement).

Colonies of Ins1Cre (*60*) GCaMP6f^f/f^ (Jackson Laboratory, stock no. 028865) on a C57BL/6J background were fed a regular chow diet and maintained under controlled temperature (21° to 23°C), humidity (45 to 50%), and light (12-hour day-night cycle) (*11*). All experimental manipulations were approved by the local ethical committee (CRCHUM, Montreal CIPA 2022–10,040 CM21022GRs).

### Generation of transgenic lines

For the construction of the *Tg(ins:eNPHR3.0-mCherry;cryaa:CFP)*, we used polymerase chain reaction (PCR) amplification with primers designed to introduce 5′ Eco RI and 3′ Pac I restriction enzyme sites flanking the cDNAs for eNPHR3.0-mCherry (Halo3-mCherry; Addgene, 59327). A previously established plasmid backbone containing ins:mKO2-zCdt1; cryaa:CFP (*59*) was digested with Eco RI/Pac I, and eNPHR3.0-mCherry was ligated using the Eco RI/Pac I sites.

For the construction of *ins:CheRiff-GFP;Myl7:GFP*, we used PCR amplification with primers designed to introduce 5′ Eco RI and 3′ Pac I restriction enzymes sites flanking the cDNA encoding CheRiff-GFP (*23*) (FCK-Optopatch2; Addgene, 51694). First, the established plasmid backbone containing ins:mKO2-zCdt1; cryaa:CFP (*59*) was digested with Kpn I to remove the Cryaa promoter and the cyan fluorescent protein (CFP). Then, we amplified the 244 minimal Myl7 zebrafish heart promoter flanked with 5′ Kpn I and 3′ Hind III (*61*); we also amplified GFP, flanked with 5′ Hind III and 3′ Spe I, and included the bGH poly(A) signal flanked with 5′ Xba I and 3′ Kpn I. We ligated the three products in a single reaction to create the plasmid containing ins:mKO2-zCdt1; Myl7:GFP. Last, the plasmid ins:mKO2-zCdt1;Myl7:GFP was digested with Eco RI/Pac I, and CheRiff-GFP was ligated using the Eco RI/Pac I sites.

To generate the *Tg(ins:CaMPARI; cryaa:RFP)* construct, the CaMPARI coding sequence was PCR-amplified from pcDNA3-CaMPARI plasmid (Addgene, plasmid no. 60421, a gift from L. Looger and E. Schreiter), adding the restriction sites Eco RI and Pac I at its 5′ and 3′ ends, respectively. The amplified PCR product and ins:Brainbow1.0 L; cryaa:mCherry vector were digested with Eco RI/Pac I. The two were ligated to yield the final construct. The final construct contains an insulin promoter driving CaMPARI and a directionally inverted eye marker cassette using a crystallin promoter (cryaa:mCherry) to select transgenic carriers. The transgenic line is abbreviated as *Tg(ins:CaMPARI)* in the manuscript.

All the constructs contain I–Sce I sites to facilitate transgenesis. Several founders were screened, and founders with Mendelian segregation were selected for each transgenic line. These lines were used in all further experiments.

### Chemical compounds

Stock solutions of 4-DP hydrochloride (Sigma-Aldrich) and GT (Sigma-Aldrich) were prepared in DMSO and stored at −20°C. Compounds were added to embryonic water (E3) containing a 1% final concentration of DMSO to be used in the treatments. The working concentration for 4-DP and for GT was 1 mM and 200 μM, respectively. Treatments were performed in six-well plates with a 10-ml final volume and up to 15 larvae per well. E3 with only 1% DMSO was used for the control treatment group. Zebrafish were treated for 2 hours before imaging.

### Live imaging and glucose injections

Transgenic zebrafish larvae were treated with 0.003% propylthiouracil (200 μM) from 1 dpf onward to inhibit pigmentation. Glucose injections and live imaging were performed as previously described using 4- to 5-dpf larvae (*11*).

### Analysis of Ca^2+^ dynamics

#### Time of response T_25_

The cumulative population response of β cells was quantified from all imaged cells. The islet area was delimited manually using the ROI (*62*) Manager in Fiji (https://imagej.net/Fiji). Using the ROI, the fluorescence intensity of GCaMP6s or K-GECO was extracted. The time of response was defined as the time required for the fluorescence of the calcium indicator (GCaMP6s or K-GECO) to show an increase of >25% of the maximum intensity after the glucose bolus or simply *T*_25_. First-responder cells were identified as those cells with the fastest average *T*_25_ from three consecutive stimulations. The fluorescence intensity was normalized using the following formula

$$\left(F_{T}-F_{\text{MIN}}\right)/\left(F_{\text{MAX}}-F_{\text{MIN}}\right)$$

where *F_T_* is the fluorescence intensity at a given time, while *F*_MAX_ and *F*_MIN_ are the maximum and minimum values recorded during the live imaging session, respectively. This normalization allows for direct comparison among islets from different animals, as they will emit varying levels of fluorescent intensities depending on the focal plane and the cell’s or islet’s accessibility to imaging. This normalization was applied to the GCaMP6s and K-GECO experiments to calculate the time of response.

#### Strength of response

The strength of response was defined as the AUC for all imaged cells after stimulation. In this case, the maximum value (*F*_MAX_) was not used for normalization because such normalization could mask a weak or null response. Instead, we only subtracted the background signal using the following formula

$$\left(F_{T}-F_{\text{MIN}}\right)/F_{\text{MIN}}$$

This method allowed to distinguish strong, weak, or null responses without forcing a maximum value.

#### Peak-amplitude and peak-width analysis

Florescence traces were normalized using the formula

$$\left(F_{T}-F_{\text{MIN}}\right)/F_{\text{MIN}}$$

For calculating the first-phase response, the peak amplitude was defined as the maximum value (*F*_MAX_) recorded during the initial rapid increase upon glucose stimulation. For the second-phase response, the peak amplitude was defined as the average of the maximum values recorded for each calcium peak. Similarly, for the first-phase response, the peak width was defined as the time spent as active, i.e., exhibiting a fluorescence intensity of >25% over the baseline. For the second-phase response, the peak width was defined as the average of the time spent as active, i.e., showing a >25% deflection over the baseline for each calcium peak.

#### Ca^2+^ imaging and RNAscope analysis

First, 3D Ca^2+^ imaging was performed using Leica SP8 MP confocal microscope, with an active resonant scanner, 5-μm *z*-intervals, five to seve *z*-slices per islet, and 1-Hz frame acquisition speed. Baseline fluorescence was recorded in each movie for 10 frames (10 s), and then the glucose stimulation was applied. Samples were paraformaldehyde (PFA)–fixed immediately after live imaging, followed by RNAscope. The whole-mount RNAscope mRNA in situ hybridization was performed following the protocol from Gross-Thebing *et al. (*63*)* with small modifications. Briefly, zebrafish larvae were fixed in 4% PFA (Thermo Fisher Scientific) for 1 hour at room temperature (RT). The larvae were washed three times in 0.1% PBT [Tween 20 in phosphate-buffered saline (PBS)] before and after the skin over the pancreas was carefully removed in each larva. The samples were dehydrated with serial washes in increasing MeOH concentrations (25%, 50%, 75%, and 2× 100%) in 0.1% PBT and stored at −20°C for at least one night.

The RNAscope Multiplex Fluorescence Assay V2 Kit [Advanced Cell Diagnostics (ACD)] was used for the assay. Samples were rehydrated by washing two times with 50% MeOH in 0.1% PBT followed by washing three times in 0.1%PBT. They were treated with protease III (ACD) for 20 min at RT followed by washing three times in 0.01% PBT. Samples were incubated with the hybridization mix containing the target probe at 40°C in HybEZ Oven (ACD) overnight. RNAscope Probe- Dr-pnpo-C3 (ref. 1169341-C3), designed by ACD, was used to target zebrafish pnpo mRNA, and 3-plex Negative Control Probe (ACD) that targets the bacterial DapB gene was used as a negative control. The hybridization steps for signal amplification and the horseradish peroxidase development were performed as described in the manufacturer’s protocol at 40°C in HybEZ II Oven. Between each incubation, samples were washed three times with 0.2× SSCT (0.01% Tween-20 in 0.2X saline-sodium citrate buffer) for 15 min each at RT. Tyramide signal amplification (TSA) Vivid 650 (Tocris) was used as the fluorophore. An additional fixation step was performed at the end of the RNAscope assay with 4% PFA for 30 min at RT. RNAscope protocol was followed by immunostaining.

Samples were washed three times (with 0.4% Triton X-100 in PBS) and blocked (in 4% bovine serum albumin and 0.4% Triton X-100 PBS) for 1 hour at RT. Primary antibody incubations were performed overnight at 4°C, and secondary antibody incubations were done for 2 hours at RT. 4′,6-Diamidino-2-phenylindole counterstaining was applied together with secondary antibody incubation. Antibodies used were anti-insulin (rabbit, GeneTex, 1:200) and Alexa Fluor 568 anti-rabbit (donkey, Invitrogen, 1:500). Samples were mounted in Vectashield (VWR) and imaged using an LSM 780 Zeiss confocal microscope using *z*-stacks. ImageJ was used for image processing and quantifications. Imaris was used to match in vivo images to RNAscope images using the nuclear mCerulean signal in β cells.

Imaris (Bitplane) and ImageJ software were used together for the manual matching the position of β cells in vivo and in the RNAscope image of each islet. First, the 3D projection of an RNAscope image was aligned manually to the in vivo image on Imaris. This was done by orienting the image on Imaris, using the nuclear mCerulean signal of β cells in each image as the guideline. This orientation information was applied to the original RNAscope image on ImageJ and saved, to be used in the following segmentation and quantification steps. ImageJ ROI manager was used for manual segmentation of β cells using the insulin antibody staining and counting transcripts using the RNAScope signal. After the segmentation of β cells in the aligned RNAscope image and the in vivo image, cells were matched manually between images. For the comparison of the average number of transcripts in the follower and the leader cells, the transcript count in each cell was normalized with the average number of transcripts per cell of the islet.

#### Murine islet isolation

Male mice aged between 8 and 16 weeks were euthanized, and collagenase solution dissolved in PBS (1 mg/ml) was delivered to the pancreas through the common bile duct. After inflation, the pancreas was dissected and digested for 12 min at 37°C. The islets were subjected to separation by gradient centrifugation with Histopaque at 1.083 and 1.119 g/ml. Isolated islets were recovered and cultured at 37°C in a humidified incubator with 5% CO_2_, in RPMI 1640 (Gibco) medium with 11 mM glucose and supplemented with penicillin (100 IU/ml), streptomycin (100 μg/ml), and fetal bovine serum (FBS; 10% v/v). One hour before imaging, the islets were transferred from the culture medium to a Krebs buffer [130 mM NaCl, 3.6 mM KCl, 1.5 mM CaCl_2_, 0.5 mM MgSO_4_, 0.5 mM NaH_2_PO_4_, 24 mM NaHCO_3_, and 10 mM Hepes (pH 7.4)] containing 3 mM glucose (*10*). For control islets, the Krebs buffer was added with 1% DMSO. For 4-DP treatment, islets were transferred to Krebs buffer with 1% DMSO and 0.5, 1, and 2 mM 4-DP.

#### Murine islet calcium imaging

Calcium imaging was performed in a Zeiss LSM 980 with Airyscan 2 super-resolution confocal microscope essentially as described earlier (*10*). The microscope was notably equipped with an incubation system set at 37°C. The laser line of 488 nm was used for GCamP6f excitation. The light was detected with a GaAsP-PMT detector with a set detection window of 493 to 540 nm. For single-cell resolution high-speed calcium islet imaging, a 40×/1.3 Apochromat oil immersion objective was used with a frame rate of 0.5 s with 0.24 μm per pixel [512 × 512 pixels (px)]. For multi-islet imaging, a 2.5×/0.085 M27 EC Plan-Neofluar objective was used with a frame rate of 2 s with 2.496 μm per pixel (2048 × 2048 px). The first 3 min of imaging corresponds to the baseline when the islets are exposed to 3 mM glucose. Subsequently, glucose is elevated to 16 mM for 33 min. Last, during the last 3 min, 30 mM KCl is delivered to force islet membrane depolarization. The AUC of GCaMP6f fluorescence was calculated by covering 3, 10, 15, and 20 min of the recordings. The AUC for KCl was calculated by covering the last 3 min after the addition of KCl.

#### Optogenetic single-cell interrogation

To perform single-cell optogenetic interrogation, we used the ROI scan technology to shine light on individual cells while recording glucose-stimulated calcium influx. The ROI scan is used for fluorescence recovery after photobleaching experiments in which an ROI is scanned with a particular laser light and intensity. Briefly, the larvae were imaged with a Leica SP8 MP confocal microscope, equipped with an ROI scan and an 25×/0.95 N.A objective. A ROI was selected encompassing the center of the nucleus with an approximate diameter of 10 μm. A baseline for K-GECO1 signal was acquired in the first 7.5 s or (50 frames) before the optogenetic interrogation was performed. The cell of interest was then exposed to a one-photon laser with 5% laser power (λ = 470 nm) for about 45 s (300 frames) while recording the Calcium dynamic simultaneously. After the laser was turned off, another 22.5 s (150 frames) were recorded. We defined optogenetic co-activation when we detected persistent calcium influx outside of the area used for illumination, i.e., the cells presented a sustained ≥40% increase of K-GECO1 signal over the background.

#### Ex vivo photoconversion of ins:CaMPARI islets and FACS

The primary islets from 4 months postfertilization *Tg(ins:CaMPARI)* animals were dissected from the fish after euthanasia. Each islet was placed in a well of 24-well plate with 5 ml of Dulbecco's Phosphate-Buffered Saline (DPBS) medium supplemented with calcium and magnesium (Thermo Fisher Scientific, 14040141), along with the appropriate concentration of d-glucose. The islets were allowed to equilibrate for 5 min. Subsequently, the entire 24-well plate was photoconverted using a 405-nm light-emitting diode (LED) array (Loctite, 1167593) for 3 s. The LED array evenly illuminated the entire dish with an intensity of 400 mW/cm^2^ and was triggered using a foot pedal switch. An internal timer using an LED controller (Loctite, 1359255) was used for precise timing of light exposure.

The photoconverted islets were dissociated into single cells by incubation in TrypLE (Thermo Fisher Scientific, 12563029) at 37°C in a benchtop shaker set at 450 rpm for 45 min. Following dissociation, TrypLE was inactivated with 10% FBS, and the cells were pelleted by centrifugation at 500*g* for 10 min at 4°C. The supernatant was carefully discarded, and the pellet resuspended in 500 μl of Hanks’ balanced salt solution (without Ca^2+^ and Mg^2+^). To remove debris, the solution was passed over a 30-μm cell filter (Miltenyi Biotec, 130-041-407). To remove dead cells, calcein violet (Thermo Fisher Scientific, C34858) was added at a final concentration of 1 μM, and the cell suspension was incubated at RT for 20 min. The single-cell preparation was sorted through a 100-μm nozzle. The following channels were used in FACS: Pacific Blue for calcein violet, fluorescein isothiocyanate (FITC) for green fluorescence, and phycoerythrin (PE) for red fluorescence.

The ex vivo experiment was carried out at four different d-glucose concentrations: 1, 2.5, 5.5, and 16.5 mM. As controls, islets incubated at 16.5 mM d-glucose but not exposed to violet light were used. Following FACS, quantification was performed for the proportion of β cells undergoing photoconversion for the four different d-glucose concentrations and control. For this, only alive cells were considered (calcein^+^). A plot was generated for the fluorescence intensity of red (PE) versus green (FITC) fluorescence. On the plot, two gates were drawn: a gate for (green^+^ and red^−^) cells and a gate for (green^+^ and red^+^) cells. The former represents the β cells that did not undergo photoconversion, and the latter represents photoconverted cells. Total β cells in the analysis were calculated as the sum of the two groups. The ratio between photoconverted β cells to total β cells represented the proportion of photoconversion. For each condition, four biological replicates were generated. Each biological replicate was generated by mixing cells from three islets. No mixing between biological replicates was performed. Animals were chosen at random for islet isolation.

#### Single-cell RNA sequencing and analysis

For single-cell RNA sequencing, β cells isolated from *Tg(ins:CaMPARI)* islets were subjected to profiling using Smart-Seq2 protocol from Takara. For this, islets were isolated, photoconverted ex vivo, dissociated, and FACS-sorted as described above. β Cells were collected from two conditions: responsive β cells displaying photoconversion at 2.5 mM and nonresponsive β cells without photoconversion at 16.5 mM. Photoconversion was defined as a high red-to-green ratio. For FACS of each condition, index sorting was used, and the cells were collected in a 96-well plate containing lysis buffer [2 μl of nuclease-free water with 0.2% Triton X-100 and 4 U of murine RNase inhibitor (New England Biolabs)]. For each condition, a total of 93 cells were collected for sequencing. Preparation of cDNA library and sequencing was performed exactly as described in a previous publication (*64*).

Raw reads in fastq format were trimmed using trim-galore with default parameters to remove adapter sequences. Trimmed reads were aligned to the zebrafish genome, GRCz10, using HISAT2 (*65*) with default parameters. htseq-count (*66*) was used to assign reads to exons, thus eventually getting counts per gene. For analysis, cells with more than 800 detected genes were used. The transcriptional profile of responsive and nonresponsive was compared using reproducibility-optimized test statistic (ROTS) (*67*) with default parameters. Genes with a false discovery rate less than 0.05 were considered as differentially expressed. For GO analysis of differentially expressed genes, FishEnrichR (*68*) was used. As background, genes expressed in at least 5% of the cells were used.

#### Transcriptomic analysis for pnpo/PNPO-expressing cells in zebrafish and human

To identify the signature of *pnpo/PNPO*-expressing cells in zebrafish and humans, we evaluated the single-cell data from zebrafish (*11*) and normoglycemic human from the data portal of HPAP (https://hpap.pmacs.upenn.edu/). The data were analyzed in RStudio using Seurat package. Unsupervised clustering was performed, and clusters were annotated using the expression of marker genes. The β cells were subsetted, and, within this group of β cells, the intersection between *pnpo* and *ins* expression was identified as *pnpo*^+^ β cells (*pnpo* raw counts of >0). Wilcoxon’s rank sum test was used to identify the differentially expressed genes between *pnpo^+^* and *pnpo^−^* cells. The up-regulated genes in *pnpo^+^* β cells (*P* < 0.05) were used for GO term and KEGG pathway analysis using the FishEnrichr tool. *PNPO* expression in human β cells from nondiabetic humans was analyzed in a similar manner (*PNPO* raw count of >0). The differentially expressed genes were identified using Wilcoxon’s rank sum test. The up-regulated genes in *PNPO^+^* β cells (*P* < 0.05) were used for GO term and KEGG pathway analysis using the FishEnrichr tool.

#### Pearson R–based connectivity and correlation analyses

Correlation analyses in an imaged islet were performed between β cell pairs and their extracted fluorescent Ca^2+^ traces over time in MATLAB using a custom-made script, as previously described (*11*, *46*). A noise reduction function (effectively a rolling average) was applied to smooth noisy data and minimize the effects of outliers. The data window size used to calculate the moving averages was set to 5% of the total data points collected during each capture. The correlation coefficient *R* between all possible (smoothed) β cell pair combinations (excluding the autocorrelation) was assessed using Pearson’s correlation. The Cartesian coordinates of the imaged cells were then incorporated in the construction of connectivity line maps. Cell pairs were connected with a straight line, the color of which represented the correlation strength and was assigned to a color-coded light-dark ramp [*R* = 0.1 to 0.25 (blue), *R* = 0.26 to 0.5 (green), *R* = 0.51 to 0.75 (yellow), and *R* = 0.76 to 1.0 (red)]. Data are also displayed as heatmap matrices, indicating all possible β cell pair connections on each axis (min = 0 and max = 1). The positive *R* values (excluding the auto-correlated cells) and the percentage of cells that were connected were averaged and compared between groups.

#### Statistical analysis

Statistical significance between two conditions was assessed using paired or unpaired Student’s *t* test using GraphPad Prism 9.0. Interactions between multiple conditions were determined using one- or two-way analysis of variance (ANOVA; with Tukey’s post hoc tests). Analyses were performed using GraphPad Prism (GraphPad software version 7.0), and significant *P* values are described in each relevant section. Values are plotted as means ± SD unless otherwise stated. Pearson *R*–based connectivity and correlation analyses and correlation analyses in an imaged islet were performed as previously described (*11*).
