# Supplementary material for: Optogenetic β cell interrogation in vivo reveals a functional hierarchy directing the Ca2+ response to glucose supported by vitamin B6
Source: Sci Adv. 2024 Jun 26;10(26):eado4513. doi: 10.1126/sciadv.ado4513 (PMC11204215; doi:10.1126/sciadv.ado4513)
Supplement: Supplementary file 1 — Figs. S1 to S10 Table S1 Legends for movies S1 to S23 Legends for data S1 and S2 [file sciadv.ado4513_sm.pdf]

Science Advances
AAAS

Supplementary Materials for

Optogenetic β cell interrogation in vivo reveals a functional hierarchy directing the Ca^{2+} response to glucose supported by vitamin B6

Luis Fernando Delgadillo-Silva *et al.*

Corresponding author: Nikolay Ninov, nikolay.ninov@tu-dresden.de; Guy A. Rutter, guy.rutter@umontreal.ca

Sci. Adv. **10**, eado4513 (2024)
DOI: 10.1126/sciadv.ado4513

The PDF file includes:

Figs. S1 to S10
Table S1
Legends for movies S1 to S23
Legends for data S1 and S2

Other Supplementary Material for this manuscript includes the following:

Movies S1 to S23
Data S1 and S2

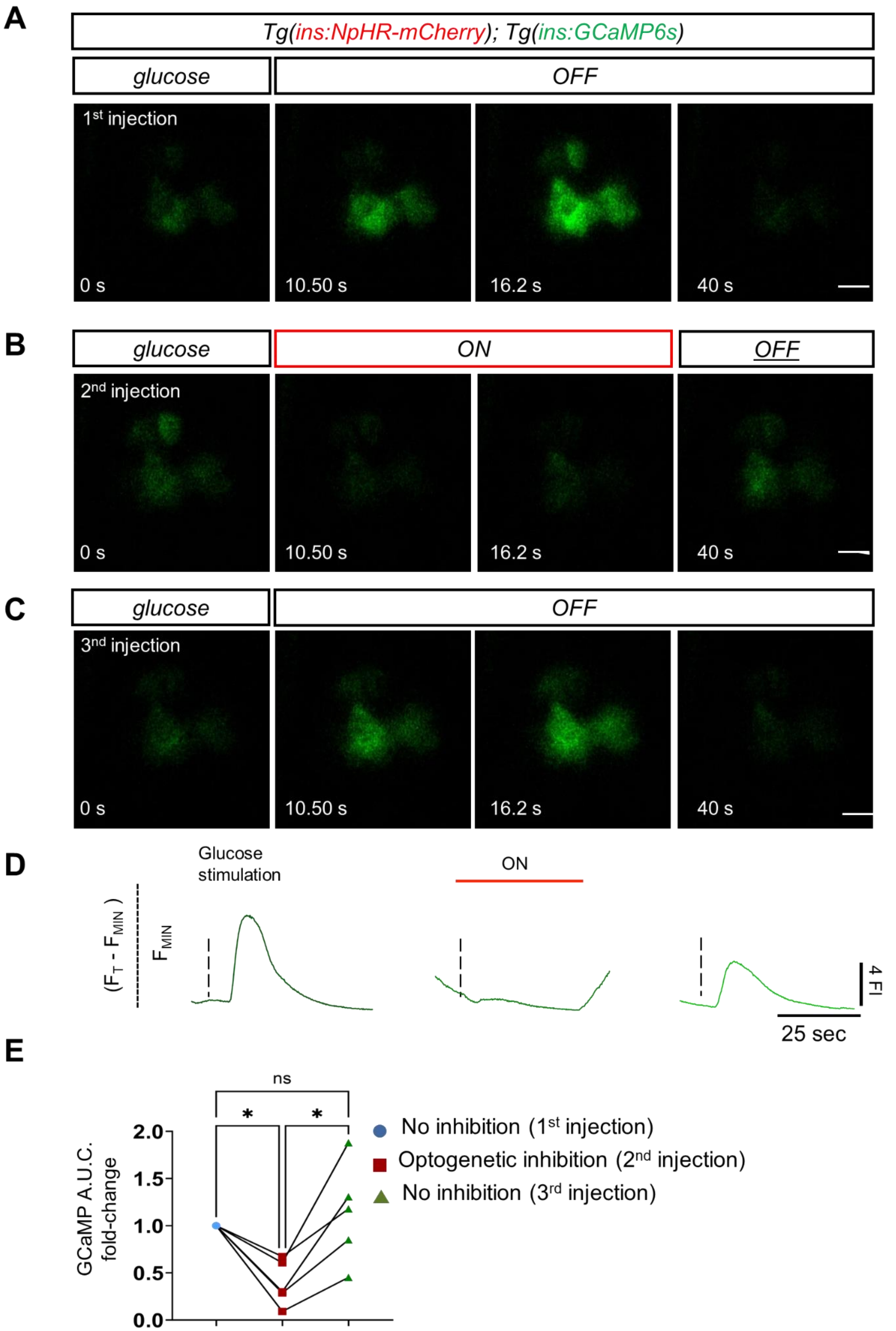

A
Tg(ins:NpHR-mCherry); Tg(ins:GCaMP6s)
glucose
OFF
1st injection
0 s
10.50 s
16.2 s
40 s
B
glucose
ON
OFF
2nd injection
0 s
10.50 s
16.2 s
40 s
C
glucose
OFF
3nd injection
0 s
10.50 s
16.2 s
40 s
D
Glucose stimulation
ON
(F_T - F_MIN) / F_MIN
4 FI
25 sec
E
ns
*
*
GCaMP A.U.C. fold-change
2.0
1.5
1.0
0.5
0.0
No inhibition (1st injection)
Optogenetic inhibition (2nd injection)
No inhibition (3rd injection)

Fig. S1. ***In vivo* optogenetic application of NpHR3.0 decreases glucose stimulated calcium influx.** **(A)** Images from the time-lapse recording at 6Hz of the islet before and after glucose stimulation in *Tg(ins:GCaMP6s); Tg(ins:eNpHR3.0-mCherry)* double-transgenic larvae. **(B)** Images from the islet shown in "A", during the second glucose injection and simultaneous *in vivo* optogenetic inhibition with a green laser ($\lambda = 561$). The optogenetic inhibition was performed by activating the laser after 7s of imaging while the glucose stimulation was delivered at 7.5s. The optogenetic inhibition was stopped after 30s of constant laser illumination. **(C)** Images from the time-lapse recording of the islet during the third glucose stimulation without optogenetic inhibition. **(D)** Traces show the normalized GCaMP6 fluorescence traces after glucose injection under normal conditions and upon light-mediated inhibition of β-cell depolarization. The red bar indicates the period of green laser exposure. **(E)** Quantification of the AUC reflecting 200 frames (30 s) of normalized GCaMP6 fluorescence for each condition, expressed as a fold-change with respect to the first stimulation. Each line represents an individual islet (n = 5 independent samples). 1-way paired ANOVA, Tukey's correction; No inhibition (1st injection) vs Optogenetic inhibition (2nd injection), * $p = 0.0109$. Optogenetic inhibition (2nd injection) vs No inhibition (3rd injection), * $p = 0.0267$. Each data point represents an individual sample. Scale bar, 10 μm.

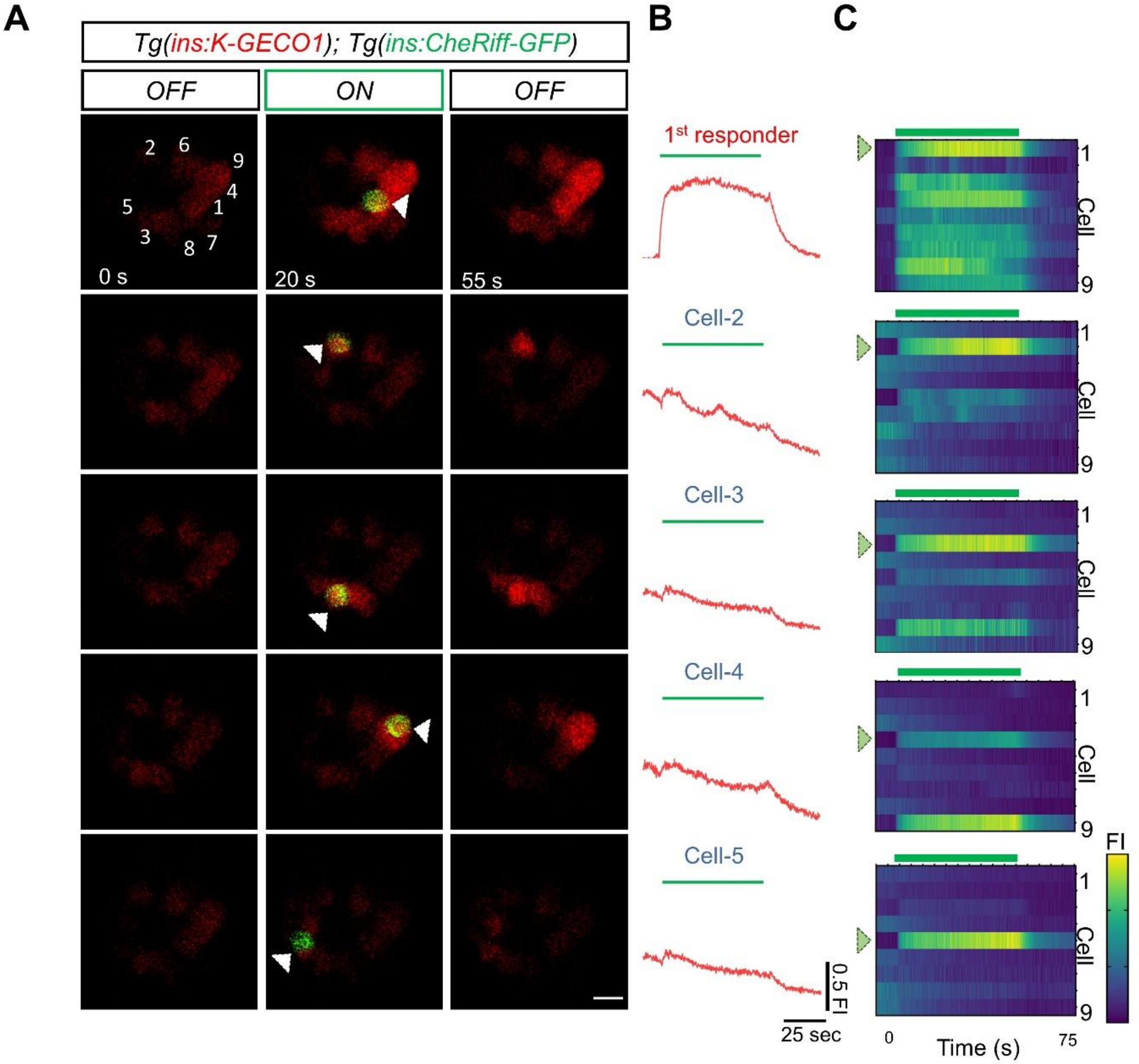

D

Cell	Average time of response	% of co-activated cells
1	24.35	0.875
2	26.9	0.125
3	30.4	0.375
4	41.7	0.125
5	59.65	0

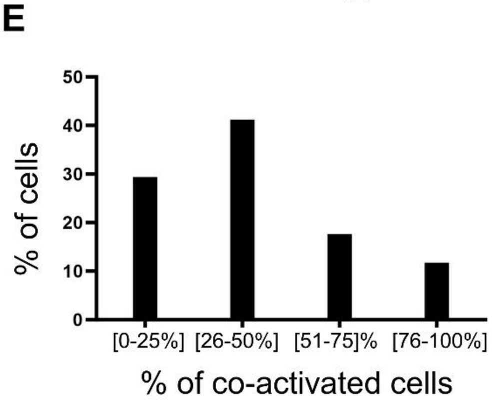

Fig. S2. ***In vivo*** **CheRiff temporal activation of first-responder β-cells propagates calcium signals across the islet.** **(A)** Images from the time-lapse recording at 6Hz of an islet before, during and after the optogenetic activation with blue laser (λ = 470) of individual cells. The images belong to the experiment shown in Figure 4. The optogenetic activation was performed using a ROI-scan encompassing the area of one follower or first-responder β-cell. The numbers indicate the order in which individual cells were activated. **(B)** The traces show the normalized K-GECO1 fluorescence traces and the peak in calcium influx after the light-mediated activation of followers and first-responder β-cell. The blue bar indicates the period of green laser exposure. **(C)** Raster plots showing the normalized K-GECO1 signal for individual cells. The green arrowhead indicated the targeted cell. **(D)** The table shows the average time of response for individual β-cells after the glucose injection (K-GECO1 signal increase >25% above baseline, T_{25}). The third column shows the percentage of β-cells that were co-activated by the illuminated β-cells. **(E)** Chart showing the proportion of β-cells grouped according to the percentage of co-activated cells (n = 39 cells from 7 independent samples). Only ~11% of β-cells can propagate the calcium across most of the cells (>75% of the cells in the plane). Scale bar, 10 μm.

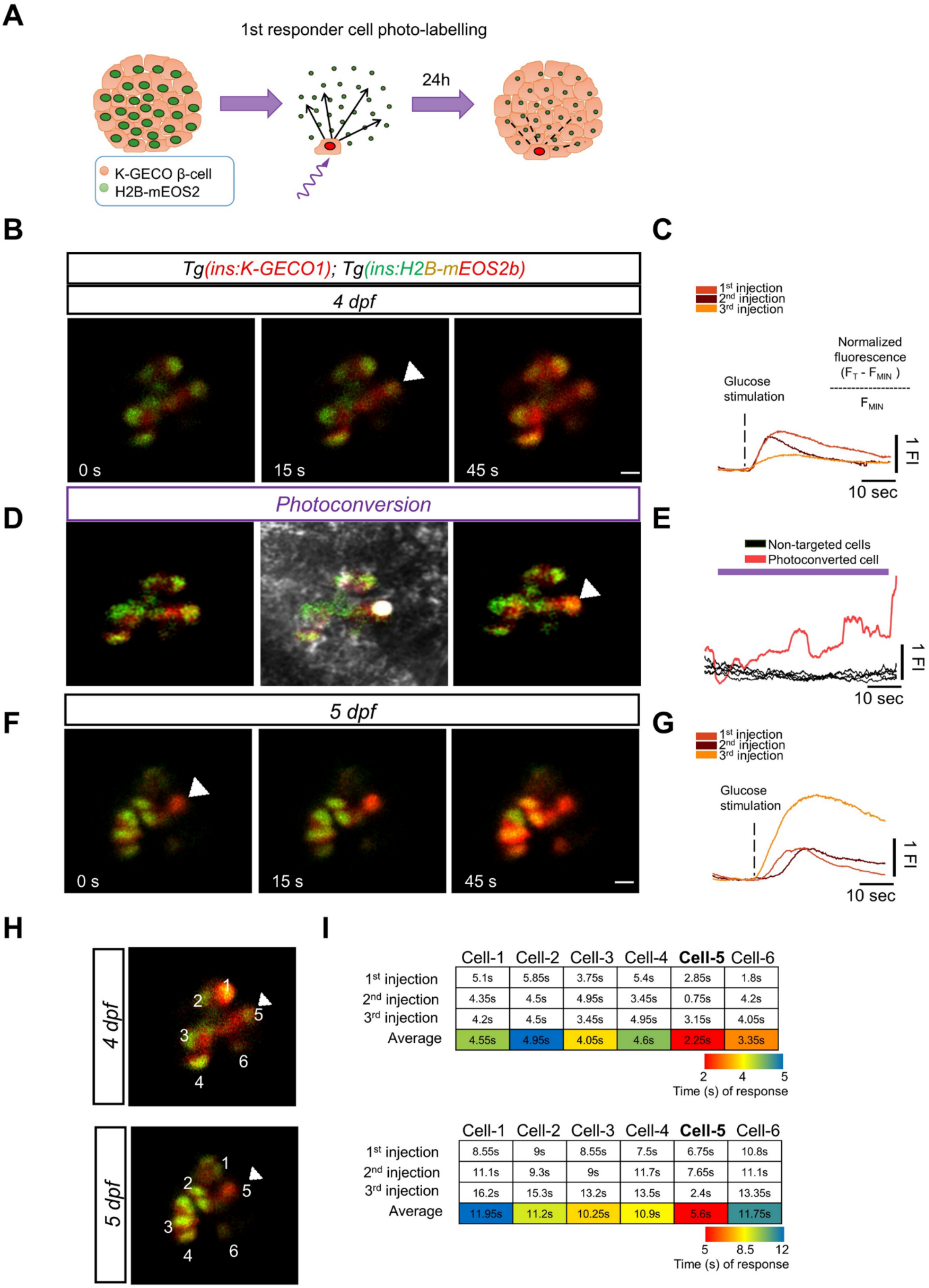
A
1st responder cell photo-labelling
24h
K-GECO β-cell
H2B-mEOS2
B
Tg(ins:K-GECO1); Tg(ins:H2B-mEOS2b)
4 dpf
0 s
15 s
45 s
C
1st injection
2nd injection
3rd injection
Normalized fluorescence
(FT - FMIN)
FMIN
Glucose stimulation
1 FI
10 sec
D
Photoconversion
E
Non-targeted cells
Photoconverted cell
1 FI
10 sec
F
5 dpf
0 s
15 s
45 s
G
1st injection
2nd injection
3rd injection
Glucose stimulation
1 FI
10 sec
H
4 dpf
5 dpf
1
2
3
4
5
6
I
Cell-1 Cell-2 Cell-3 Cell-4 Cell-5 Cell-6
1st injection 5.1s 5.85s 3.75s 5.4s 2.85s 1.8s
2nd injection 4.35s 4.5s 4.95s 3.45s 0.75s 4.2s
3rd injection 4.2s 4.5s 3.45s 4.95s 3.15s 4.05s
Average 4.55s 4.95s 4.05s 4.6s 2.25s 3.35s
2 4 5
Time (s) of response
Cell-1 Cell-2 Cell-3 Cell-4 Cell-5 Cell-6
1st injection 8.55s 9s 8.55s 7.5s 6.75s 10.8s
2nd injection 11.1s 9.3s 9s 11.7s 7.65s 11.1s
3rd injection 16.2s 15.3s 13.2s 13.5s 2.4s 13.35s
Average 11.95s 11.2s 10.25s 10.9s 5.6s 11.75s
5 8.5 12
Time (s) of response

Fig. S3. Single-cell tracking *in vivo* shows that first-responder cells are stable for periods of 24h. **(A)** A cartoon showing the rationale for *in vivo* single-cell photoconversion and tracking. β-cells express K-GECO1 and the green-to-red photoconvertible mEOS2b protein fused to histone2B (H2B). After identifying the first-responder cell, the nucleus of the cell is photoconverted from green to red by shining UV-light in a single-cell. The cell is traced for 24h. **(B)** Images from the time-lapse recording at 6Hz of the islet during a glucose injection at 4 dpf. The white arrowhead indicates the first-responder β-cell. **(C)** The traces show the cumulative normalized cytoplasmic K-GECO1 fluorescence traces and the peak in calcium influx after glucose stimulation. Glucose was injected at 5-min intervals. **(D)** Images from the time-lapse recording of the islet shown in "B" during the photoconversion of the first-responder cell. The photoconversion was done with a ROI-scan encompassing the area of one-cell nucleus. The white arrowhead indicates the photo-labelled β-cell. **(E)** The traces show the ratio of red/green fluorescence during the photo-conversion of mEOS2b for individual cells. **(F)** Images from the time-lapse recording of the islet during a glucose injection at 5 dpf. The white arrowhead indicates the photo-labelled cell. **(G)** The traces show the cumulative normalized cytoplasmic K-GECO1 fluorescence traces and the peak in calcium influx after glucose stimulation. Glucose was injected at 5-min intervals. **(H)** Images of the islet shown in "B-F" with numbered cells. (**I)** Time of response for each cell after the glucose injection at 4 and 5dpf. The photo-labelled β-cell remained the first-responder cell after 24h. Scale bar = 10μm.

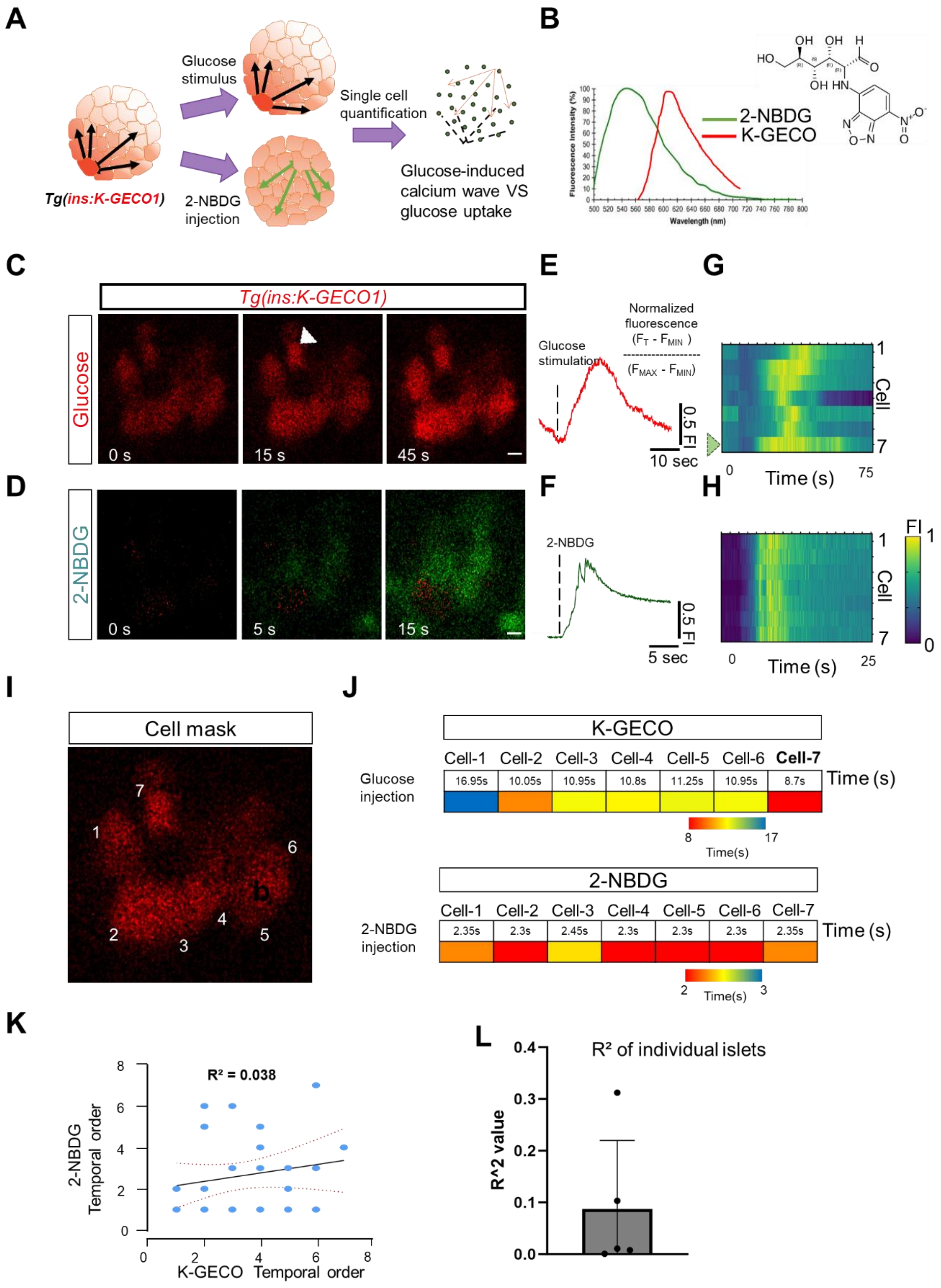
A
B
Glucose stimulus
Single cell quantification
2-NBDG injection
Tg(ins:K-GECO1)
Glucose-induced calcium wave VS glucose uptake
2-NBDG
K-GECO
Fluorescence Intensity (%)
Wavelength (nm)
C
D
E
F
G
H
Tg(ins:K-GECO1)
Glucose
2-NBDG
0 s
15 s
45 s
0 s
5 s
15 s
Glucose stimulation
Normalized fluorescence
(F_T - F_MIN)
(F_MAX - F_MIN)
0.5 FI
10 sec
5 sec
Cell
Time (s)
0
75
25
FI
1
0
7
I
J
Cell mask
K-GECO
Cell-1 Cell-2 Cell-3 Cell-4 Cell-5 Cell-6 Cell-7
Glucose injection
16.95s 10.05s 10.95s 10.8s 11.25s 10.95s 8.7s
Time (s)
8
17
Time(s)
2-NBDG
2-NBDG injection
2.35s 2.3s 2.45s 2.3s 2.3s 2.3s 2.35s
2
3
K
L
R² = 0.038
2-NBDG Temporal order
K-GECO Temporal order
R² of individual islets
R^2 value

Fig. S4. **The speed of glucose uptake does not define the first-responder cell**. **(A)** Cartoon representing the comparison between the calcium responses upon glucose stimulation versus the glucose uptake using the green fluorescent glucose analog 2-NBDG. **(B)** The fluorescence spectra of 2-NBDG and K-GECO do not overlap and can be combined in live imaging. **(C)** Snapshots from a time-lapse recording of the primary islet at 6Hz before and after glucose injection. The white arrow points to the β-cell that responded first to the glucose stimulus. **(D)** Snapshots from a time-lapse recording of the primary islet at 20Hz (50ms per frame) before and after 2-NBDG injection. The green signal corresponds to the 2-NBDG signal. **(E, F)** The traces show the normalized K-GECO1 and normalized 2-NBDG fluorescence traces for the calcium signal and the glucose uptake signal. FI, fluorescence intensity. **(G, H)** Raster plots show the normalized K-GECO1 and 2-NBDG signal for individual cells. The green arrowhead indicated the first-responder cell. **(I)** The numbers indicate the cells that were analyzed in "C" and "D" to quantify the individual time of response (defined as a > 25% increase in K-GECO and 2-NBDG fluorescence after injection). (**J**) The table shows a color key of the temporal order of response for individual cells to glucose injection (K-GECO1) and glucose uptake (2-NBDG). (**K**) Graph plotting each cell's temporal order of activation (K-GECO1) versus the glucose uptake (2-NBDG) from the islet shown in "I". The black line shows a linear regression and the associated R^2 value. The dotted red lines show the 95% confidence interval. Each dot represents one cell. (**L**) Graph showing the R^2 from 5 independent samples with an average R^2 value of 0.087 and ± SD of 0.13. (n = 5 independent samples). Data shown as mean ± SD. Scale bar = 10μm.

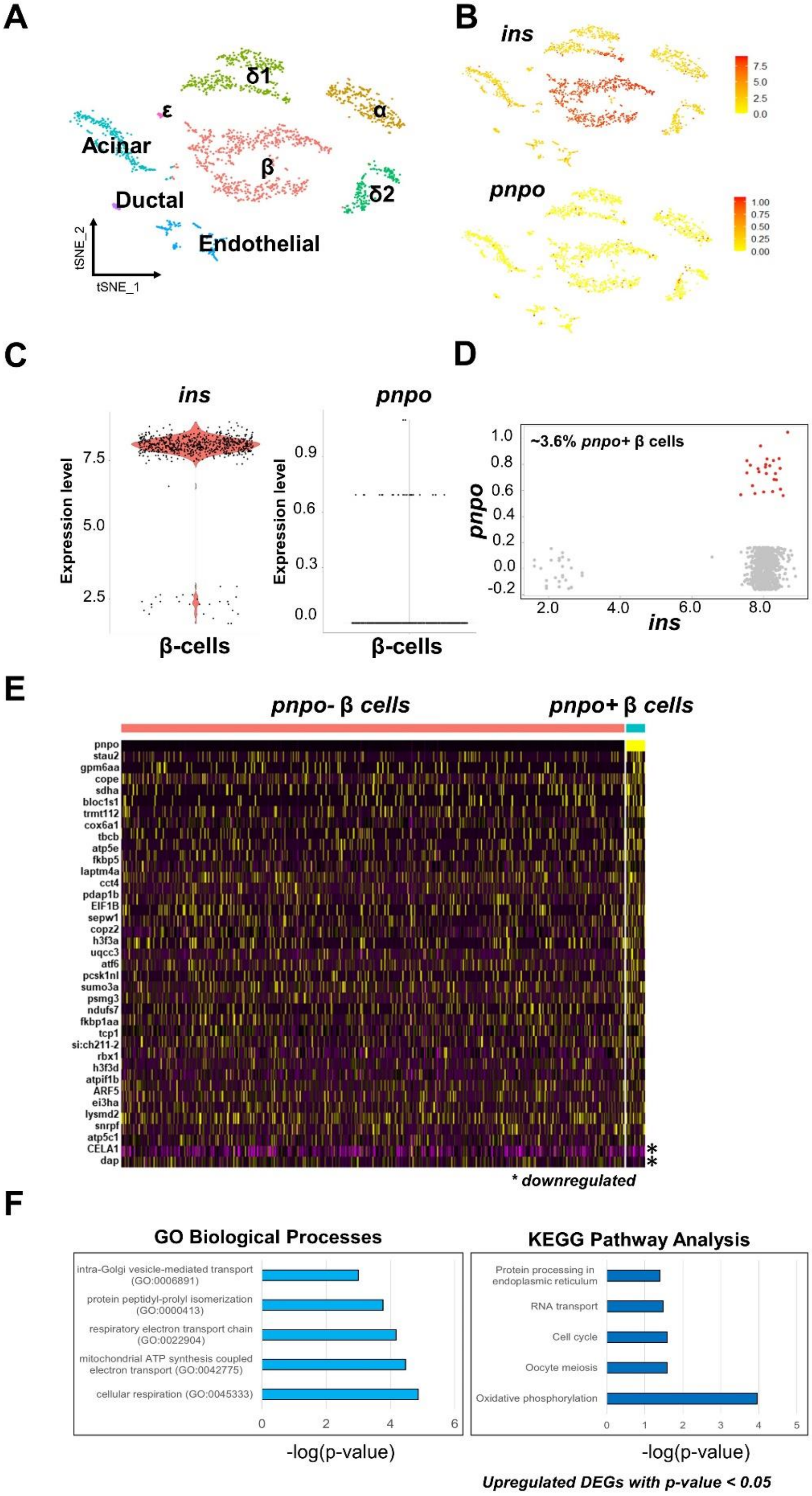

A
δ1
ε
α
Acinar
β
Ductal
δ2
Endothelial
tSNE_2
tSNE_1
B
ins
pnpo
C
ins
pnpo
Expression level
β-cells
D
~3.6% pnpo+ β cells
pnpo
ins
E
pnpo- β cells
pnpo+ β cells
pnpo
stau2
gpm6aa
cope
sdha
bloc1s1
trmt112
cox6a1
tbcb
atp5e
fkbp5
laptm4a
cct4
pdap1b
EIF1B
sepw1
copz2
h3f3a
uqcc3
atf6
pcsk1nl
sumo3a
psmg3
ndufs7
fkbp1aa
tcp1
si:ch211-2
rbx1
h3f3d
atpif1b
ARF5
ei3ha
lysmd2
snrpf
atp5c1
CELA1
dap
* downregulated
F
GO Biological Processes
intra-Golgi vesicle-mediated transport (GO:0006891)
protein peptidyl-prolyl isomerization (GO:0000413)
respiratory electron transport chain (GO:0022904)
mitochondrial ATP synthesis coupled electron transport (GO:0042775)
cellular respiration (GO:0045333)
-log(p-value)
KEGG Pathway Analysis
Protein processing in endoplasmic reticulum
RNA transport
Cell cycle
Oocyte meiosis
Oxidative phosphorylation
-log(p-value)
Upregulated DEGs with p-value < 0.05

Fig. S5. Characterization of *pnpo*$^{+}$ β- cells in zebrafish. **(A)** TSNE plot from single-cell RNA sequencing of the 2 mpf zebrafish pancreas highlighting pancreatic cell types. **(B)** Feature plots showing the expression of *ins* and *pnpo* in pancreatic cells. **(C)** Violin plot showing the log-normalized expression of *ins* and *pnpo* in β-cells. **(D)** Feature scatter plot highlighting the *ins* and *pnpo* expressing β-cells. The double-positive cells are marked in red. **(E)** Heat-map highlighting differentially expressed genes between *pnpo*- and *pnpo*+ β-cells. **(F)** Bar plots showing the Gene Ontology (GO) and KEGG pathway analysis performed using upregulated DEGs from *pnpo*+ β-cells ($p < 0.05$)

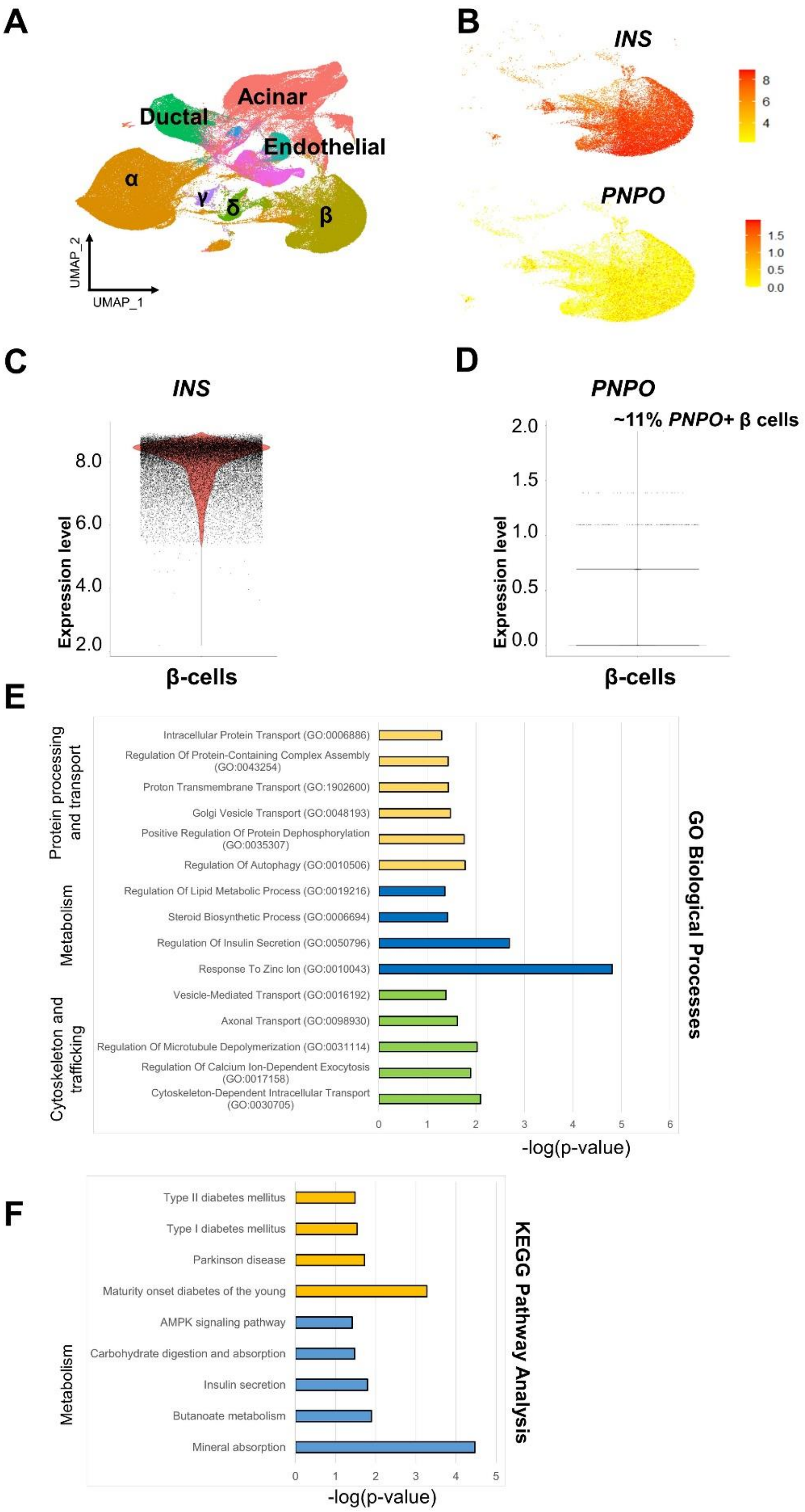
A
Acinar
Ductal
Endothelial
α
γ
δ
β
UMAP_2
UMAP_1
B
INS
8
6
4
PNPO
1.5
1.0
0.5
0.0
C
INS
8.0
6.0
4.0
2.0
Expression level
β-cells
D
PNPO
~11% PNPO+ β cells
2.0
1.5
1.0
0.5
0.0
Expression level
β-cells
E
Protein processing and transport
Metabolism
Cytoskeleton and trafficking
Intracellular Protein Transport (GO:0006886)
Regulation Of Protein-Containing Complex Assembly (GO:0043254)
Proton Transmembrane Transport (GO:1902600)
Golgi Vesicle Transport (GO:0048193)
Positive Regulation Of Protein Dephosphorylation (GO:0035307)
Regulation Of Autophagy (GO:0010506)
Regulation Of Lipid Metabolic Process (GO:0019216)
Steroid Biosynthetic Process (GO:0006694)
Regulation Of Insulin Secretion (GO:0050796)
Response To Zinc Ion (GO:0010043)
Vesicle-Mediated Transport (GO:0016192)
Axonal Transport (GO:0098930)
Regulation Of Microtubule Depolymerization (GO:0031114)
Regulation Of Calcium Ion-Dependent Exocytosis (GO:0017158)
Cytoskeleton-Dependent Intracellular Transport (GO:0030705)
0 1 2 3 4 5 6
-log(p-value)
GO Biological Processes
F
Type II diabetes mellitus
Type I diabetes mellitus
Parkinson disease
Maturity onset diabetes of the young
AMPK signaling pathway
Carbohydrate digestion and absorption
Insulin secretion
Butanoate metabolism
Mineral absorption
Metabolism
0 1 2 3 4 5
-log(p-value)
KEGG Pathway Analysis

Fig. S6. Characterization of *pnpo*$^+$ β-cells in human. **(A)** UMAP plot of single-cell RNA sequencing of pancreatic cells of healthy donors from the Human Pancreas Analysis Program (HPAP consortium). **(B)** Feature plots showing the expression of *ins* and *pnpo* in β-cells. **(C)** Violin plot showing the log-normalized expression of *ins*. **(D)** Violin plot highlighting the expression of *pnpo* in β-cells (~11%). **(E-F)** Bar plots showing the Gene Ontology (GO) and KEGG pathway analysis performed using the upregulated DEGs in *pnpo*+ β-cells ($p < 0.05$)

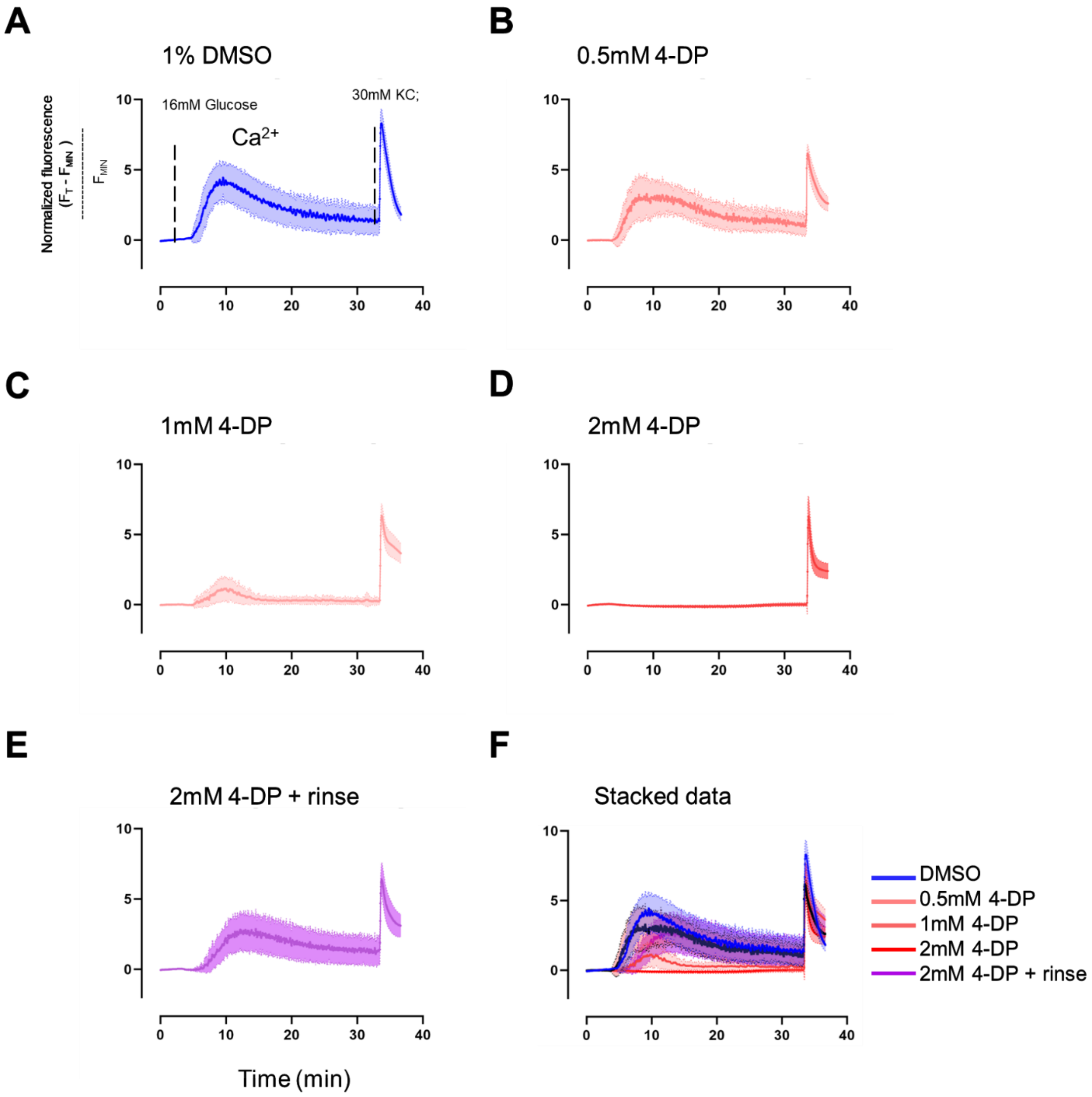

A
1% DMSO
B
0.5mM 4-DP
C
1mM 4-DP
D
2mM 4-DP
E
2mM 4-DP + rinse
F
Stacked data
16mM Glucose
30mM KCl
Ca2+
Normalized fluorescence
(FT - FMIN)
FMIN
Time (min)
DMSO
0.5mM 4-DP
1mM 4-DP
2mM 4-DP
2mM 4-DP + rinse

Fig. S7. The vitamin B6 antagonist 4-Deoxypyridoxine (4-DP) reduces calcium responses in a dose-dependent manner in mouse islets. (A-E) Fluorescence traces from *in vitro* calcium imaging of islets from C57BL/6J Ins1Cre:GCaMP6f$^{fl/fl}$ upon 1% DMSO (control) or 4-Deoxypyridoxine (4-DP) treatment. The lines represent the average GCaMP6f fluorescence traces and standard deviations from the imaged islets (n= 48 islets from 3 mice). **(B-E)** Average fluorescence traces of islets treated with different concentrations of 4-DP. The experimental groups include 1% DMSO (control), 0.5 mM 4-DP, 1 mM 4-DP, 2 mM 4-DP, and a 1 min rinse of 2 mM 4-DP (n= 48, n = 31, n = 42, n = 43, n = 37, respectively from 3 mice). **(F)** Stacked data from all the conditions.

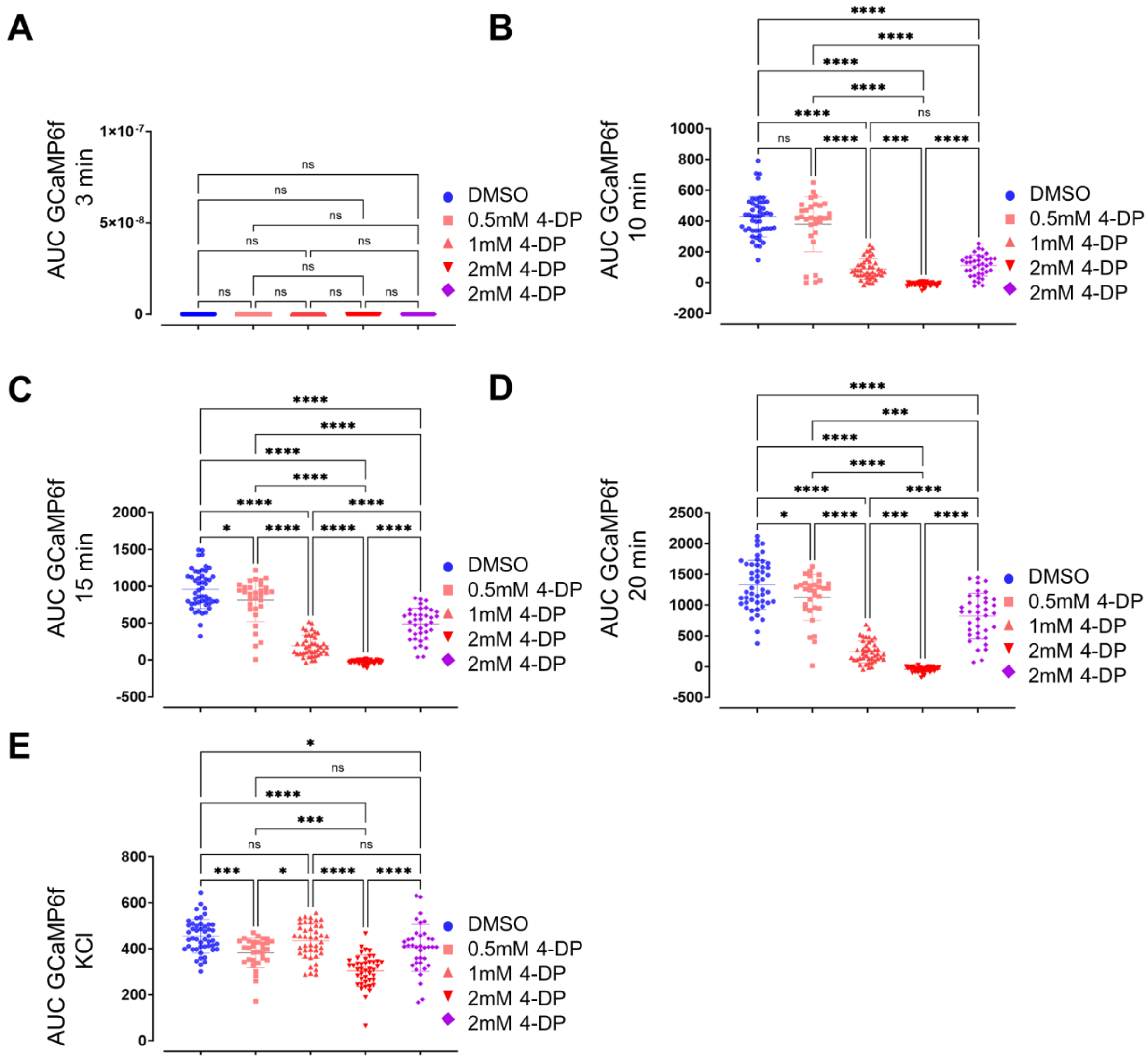
A
AUC GCaMP6f
3 min
1×10-7
5×10-8
0
ns
DMSO
0.5mM 4-DP
1mM 4-DP
2mM 4-DP
2mM 4-DP
B
AUC GCaMP6f
10 min
1000
800
600
400
200
0
-200
****
***
ns
DMSO
0.5mM 4-DP
1mM 4-DP
2mM 4-DP
2mM 4-DP
C
AUC GCaMP6f
15 min
2000
1500
1000
500
0
-500
****
*
DMSO
0.5mM 4-DP
1mM 4-DP
2mM 4-DP
2mM 4-DP
D
AUC GCaMP6f
20 min
2500
2000
1500
1000
500
0
-500
****
***
*
DMSO
0.5mM 4-DP
1mM 4-DP
2mM 4-DP
2mM 4-DP
E
AUC GCaMP6f
KCl
800
600
400
200
0
*
ns
****
***
DMSO
0.5mM 4-DP
1mM 4-DP
2mM 4-DP
2mM 4-DP

Fig. S8. Inhibition of calcium activity in response to vitamin B6 antagonism in mouse islets. (A-E) Area under the curve (AUC) quantifications of calcium activity in control and 4-DP-treated mouse islets at 3 min, 10 min, 15 min, 20 min following glucose stimulation, and after addition of KCl. Each dot represents an islet. The experimental groups include: 1% DMSO (control), 0.5 mM, 1 mM, 2 mM 4-DP, and a 1 min rinse of 2 mM 4-DP (n= 48, n = 31, n = 42, n = 43, and n = 37 islets, respectively from 3 mice). Fluorescence traces are presented in Fig. S7A–E. Data significance was analyzed by 1-way unpaired ANOVA, Tukey's correction. Panel B, *** p = 0.0002, **** p = 8.7932E-57, ns, not significant. Panel C, *p = 0.0152, **** p = 1.11298E-62. Panel D, * p = 0.0309, *** p = 0.0005, **** p = 3.75E-60. Panel E, * p = 0.0453, *** p = 0.0003, **** p = 1.81E-16. ns, not significant. Data are mean ± SD.

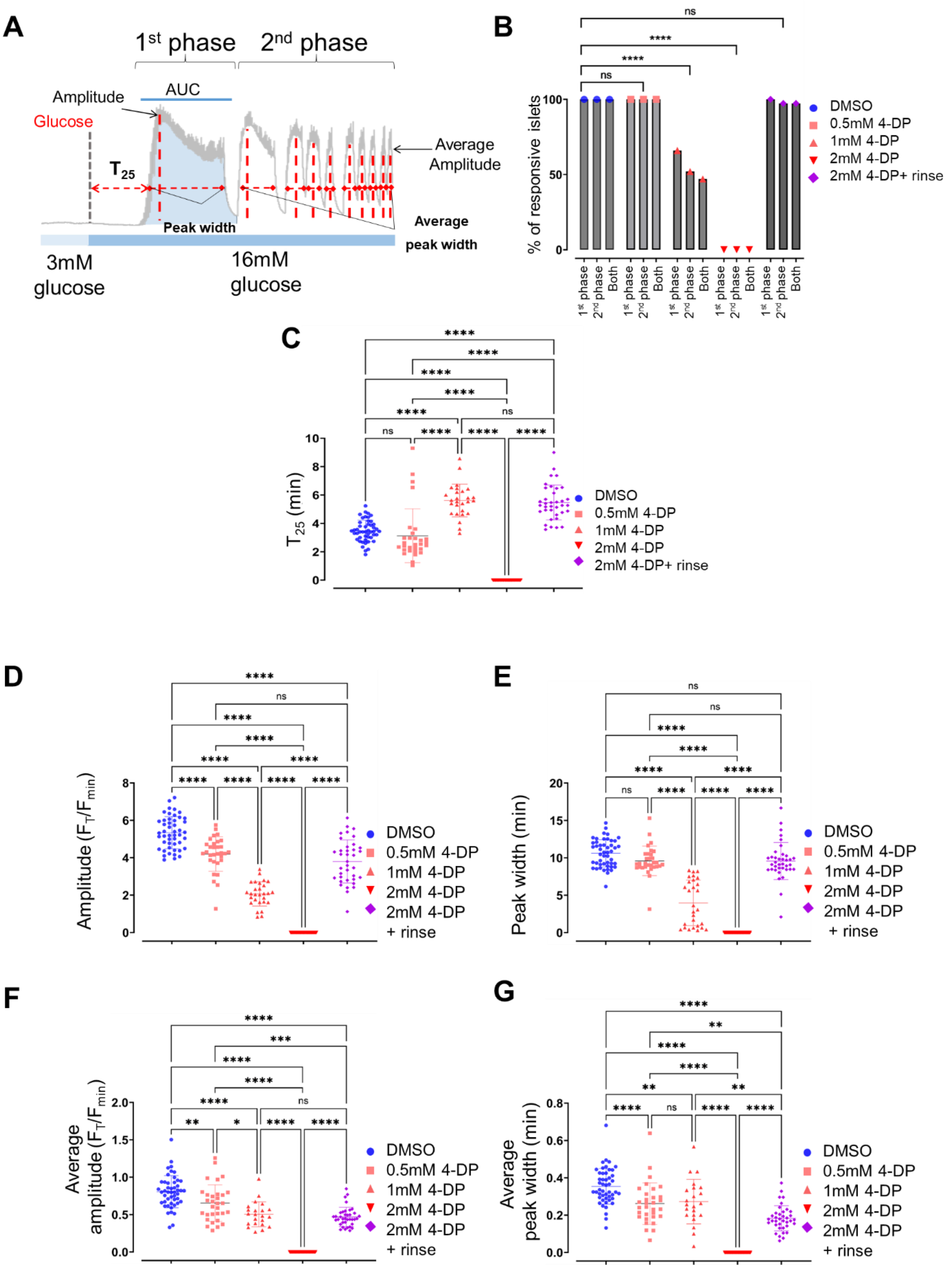

A
1st phase
2nd phase
AUC
Amplitude
Glucose
T_{25}
Average Amplitude
Peak width
Average peak width
3mM glucose
16mM glucose
B
% of responsive islets
DMSO
0.5mM 4-DP
1mM 4-DP
2mM 4-DP
2mM 4-DP+ rinse
1st phase
2nd phase
Both
ns
****
C
T_{25} (min)
D
Amplitude (F_T/F_{min})
E
Peak width (min)
F
Average amplitude (F_T/F_{min})
G
Average peak width (min)
2mM 4-DP + rinse

Fig. S9. Vitamin B6 antagonism reduces in a dose-dependent manner the first- and the second-phase responses to glucose. **(A)** Graphical representation of a typical calcium trace of mouse islets. T_{25} is the time taken from the glucose addition in the media to observing a GCAMP fluorescence increase of 25% over the baseline. The amplitude of the first-phase response is defined as the maximum value recorded during the initial increase in calcium. The peak width is defined as the time spent with a fluorescence signal greater than 25% over the baseline. During the second-phase response, the amplitudes and the peak widths are averaged. **(B)** The graph shows the percentage of islets that showed significant activity during the first-phase and the second-phase responses upon DMSO or 4-DP treatment. **(C)** T_{25} quantifications for controls and 4-DP treated islets. **(D-E)** Peak amplitude and peak width quantifications of the first-phase response of DMSO and 4-DP-treated islets. **(F-G)** Average peak amplitude and peak width quantifications of the second-phase response upon DMSO or 4-DP treatment. The experimental groups include 0.5 mM 4-DP, 1mM 4-DP, 2mM 4-DP, and a 1 min rinse of 2mM 4-DP (n = 31, n = 42, n = 43, n = 37, respectively from 3 mice). Data significance was analyzed by 1-way unpaired ANOVA, Tukey's correction. Panel B, **** p = 2.66E-10. Panel C, **** p = 5.06E-58. Panel D, **** p = 6.28E-77. Panel E, **** p =5.02E-67. Panel F, * p = 0.0158, ** p-value = 0.0015, *** p = 0.0001, **** p = 8.02E-53. Panel G, ** p = 0.0023, **** p = 4.21E-46. ns, not significant. Data are shown as mean ± SD.

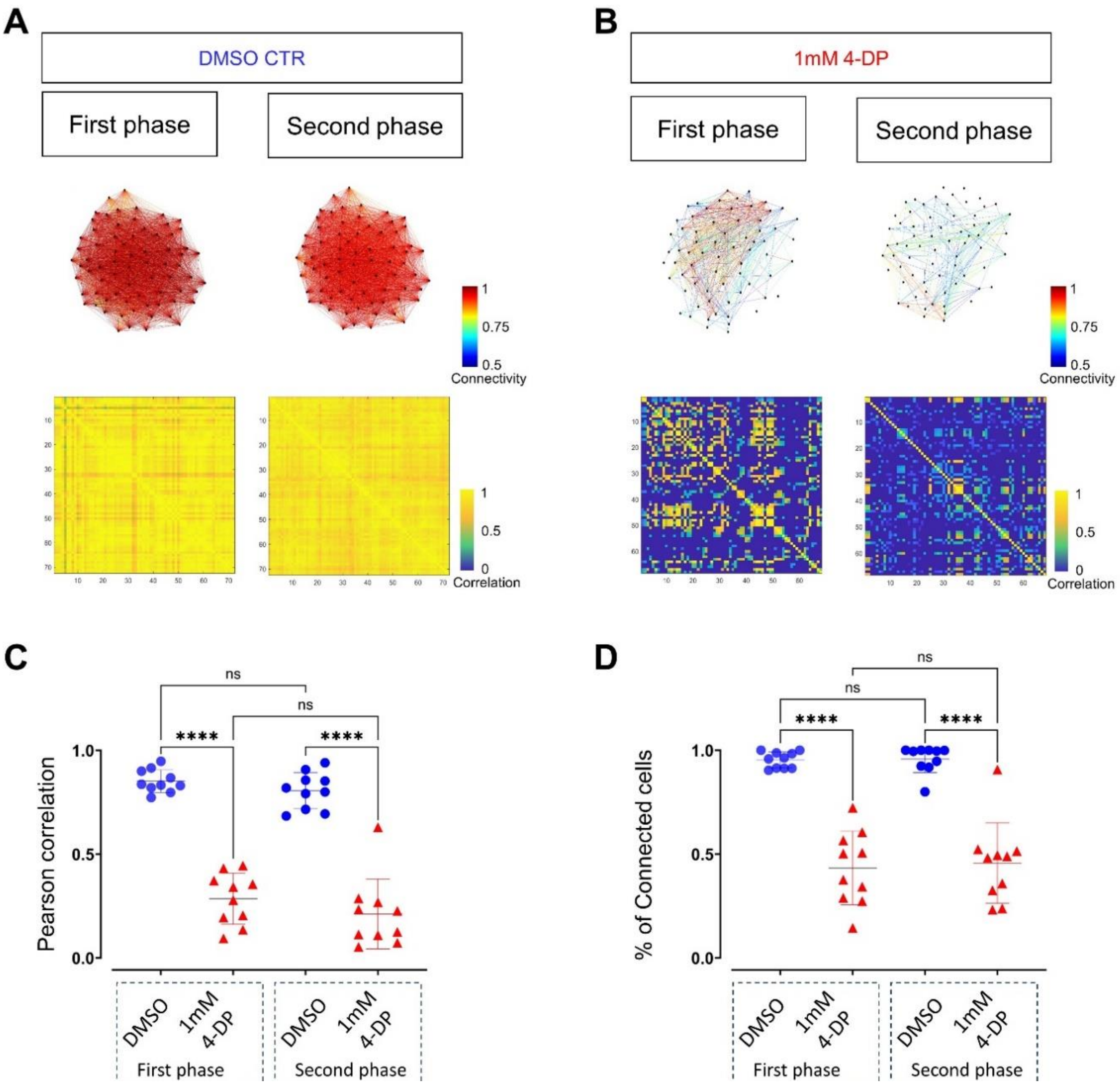

A
DMSO CTR
First phase
Second phase
Connectivity
Correlation
B
1mM 4-DP
First phase
Second phase
Connectivity
Correlation
C
Pearson correlation
ns
****
DMSO
1mM 4-DP
First phase
Second phase
D
% of Connected cells
ns
****
DMSO
1mM 4-DP
First phase
Second phase

Fig. S10. Vitamin B6 antagonism reduces β-cell coordination in mouse islets during the first and the second-phase responses to glucose. (A-B) Connectivity maps and Pearson correlation matrices of the imaged islets during the first- and the second-phase responses. **(C)** Pearson correlation of individual islets (1-way unpaired ANOVA, Tukey's correction, **** p-value = 1.73E-12, ns, not significant). **(D)** Percentage of connected cells in individual islets. 1-way unpaired ANOVA, Tukey's correction **** p = 2.71E-16, ns, not significant). n = 10 islets from 3 mice (1% DMSO) and n = 10 islets from 3 mice (1mM 4-DP). Data are mean ± SD.

Table S1. General properties of commonly used optogenetic actuators

	CHR2(1)	CHERIFF(2)	CHRIMSONR(3)	ENPHR3.0(4)
SPECIES	*Chlamydomonas reinhardtii*	*Scherffelia dubia*	*Chlamydomonas noctigama*	*Natronomonas pharaonis*
EXCITATION PEAK (Λ)	470nm	460nm	590nm	589nm
REPORTED CONDITIONS: Λ AND LIGHT INTENSITY	470nm, 19.8 mW/mm^2	488nm, 500mW/cm^2	625 nm, 3.14 mW/mm^2	593 nm, 21.7 mW/mm^2
LIGHT SENSITIVITY/EC50	~11 mW/cm^2	~22 mW/cm^2	N.R.	N.R.
PEAK CURRENT	~0.7nA	~2nA	~0.7nA	~0.74nA (outward)
DESENSITIZATION (STEADY-STATE CURRENT OVER PEAK CURRENT)	~0.22	~0.665	~0.58	N.R.
OPENING RATE $T_{(1)}$	~1.21ms	~4.5ms	~9ms	~6.1ms
CLOSING RATE $T_{(1)}$	~13.5ms	~16ms	~15.8ms	~6.9ms
ION SPECIFICITY	Cations	Cations	Cations	Cl-
COMMENTS FROM THIS STUDY	Works in combination with jRCaMP1a and K-GECO1	Works in combination with jRCaMP1a and K-GECO1	10-20% activation by 488nm laser light	Works *in vivo*, but it desensitizes in around 20s-30s of constant illumination

[1]Values obtained from reference *(22)*

[2]Values obtained from reference *(23)*

[3]Values obtained from reference *(24)*

[4]Values obtained from references *(25,26)*

Movie S1. Calcium imaging of zebrafish β-cells expressing GCaMP6s and cdt1-mCherry under the insulin promoter. The first 7.4s of the video are taken as the baseline calcium activity. The changes of GCaMP fluorescence signal correspond to the glucose-stimulated calcium influx after the 25 mM glucose injection into the circulatory system. The video shows three pulses of glucose, that were spaced by 5-min intervals.

Movie S2. Optogenetic inhibition of zebrafish β-cells during the glucose-induced influx of calcium. The β-cells express GCaMP6s and eNPHR3.0-mCherry under the insulin promoter. Three separate pulses of glucose were delivered without inhibition, with green light (λ = 561) inhibiting the whole islet, and a third time without inhibition.

Movie S3. Calcium imaging of zebrafish β-cells expressing GCaMP6s and eNPHR3.0-mCherr under the insulin promoter. A pulse of glucose was delivered without inhibition to find the first responder cells.

Movie S4. Optogenetic inhibition of a single-cell during the glucose-induced influx of calcium. A pulse of glucose was delivered with the simultaneous single-cell inhibition of a follower cell using a green light (λ = 561).

Movie S5. Optogenetic inhibition of a single-cell during the glucose-induced influx of calcium. A pulse of glucose was delivered with the simultaneous single-cell inhibition of a first responder cell using a green light (λ = 561).

Movie S6. Pan islet optogenetic activation of zebrafish β-cells expressing the red calcium indicator K-GECO1 and the blue channelrhodopsin CheRiff-GFP under the insulin promoter.

Movie S7 Optogenetic activation of a single-cell in absence of any glucose stimulus. Optogenetic single-cell activation using a blue light (λ = 470) and simultaneous recording of the calcium propagation across other cells.

Movie S8. Optogenetic activation of a single-cell in absence of any glucose stimulus. Optogenetic single-cell activation using a blue light (λ = 470) and simultaneous recording of the calcium propagation across other cells.

Movie S9. Optogenetic activation of a single-cell in absence of any glucose stimulus. Optogenetic single-cell activation using a blue light (λ = 470) and simultaneous recording of the calcium propagation across other cells.

Movie S10. Calcium imaging of zebrafish β-cells expressing K-GECO1 and CheRiff-GFP under the insulin promoter. Three separate pulses of intracardiac glucose were given and time of response for each cell was calculated.

Movie S11. Optogenetic activation of the first-responder cell in absence of any glucose stimulus using blue light (λ = 470) and simultaneous recording of the calcium propagation across other cells.

Movie S12. Optogenetic activation of 2 different follower cells in absence of any glucose stimulus using blue light (λ = 470) and simultaneous recording of the calcium propagation across other cells.

Movie S13. Calcium imaging of zebrafish β-cells expressing K-GECO1 and mEOS2b-H2B under the insulin promoter. Three separate pulses of intracardiac glucose were given and the individual time of response for each cell was calculated.

Movie S14. Photoconversion of the nuclear label mEOS2b using UV-light (λ = 405).

Movie S15. Calcium imaging of the same zebrafish 24h after the photolabelling of the first-responder β-cell. Three separate pulses of intracardiac glucose were given and the individual time of response for each cell was calculated.

Movie S16. Pre RNAScope calcium imaging of zebrafish pancreatic islets. Representative *in vivo* calcium imaging of beta cells in *Tg(ins:GCaMP6s); Tg(ins:NLS-mCerulean)* zebrafish. The movie displays 3 consecutive recordings of the same zebrafish with glucose stimulations. The first 10 s in each recording corresponds to the baseline calcium activity. The glucose stimulation is performed at 10 sec, and the activity is recorded for 2 min. Scale bar = 10 µm

Movie S17. Calcium imaging of control zebrafish pancreatic islets (DMSO.treated). Representative *in vivo* calcium imaging of beta cells in *Tg(ins:GCaMP6s); Tg(ins:cdt1-mCherry)* zebrafish after 2 h of 1% DMSO treatment. The movie displays 3 consecutive recordings of the same zebrafish with glucose stimulations. The first 10 s in each recording corresponds to the

baseline calcium activity. The glucose stimulation is performed at 10 s, and the activity is recorded for 2 min. Scale bar = 10 µm

Movie S18. Calcium imaging of 4-Deoxypyridoxine (4-DP)-treated zebrafish pancreatic islets. Representative *in vivo* calcium imaging of beta cells in *Tg(ins:GCaMP6s); Tg(ins:cdt1-mCherry)* zebrafish after 2 h of 1 mM 4-DP treatment. The movie displays 3 consecutive recordings of the same zebrafish with glucose stimulations. The first 10 s in each recording corresponds to the baseline calcium activity. The glucose stimulation is performed at 10 s, and the activity is recorded for 2 min. Scale bar = 10 µm

Movie S19. Calcium imaging of Ginkgotoxin (GT)-treated zebrafish pancreatic islets. Representative *in vivo* calcium imaging of beta cells in *Tg(ins:GCaMP6s); Tg(ins:cdt1-mCherry)* zebrafish after 2h of 200 µM GT treatment. The movie displays 3 consecutive recordings of the same zebrafish with glucose stimulations. The first 10 s in each recording corresponds to the baseline calcium activity. The glucose stimulation is performed at 10 s, and the activity is recorded for 2 min. Scale bar = 10 µm

Movie S20. Calcium imaging of wild-type control (*pnpo*$^{+/+}$) zebrafish pancreatic islets. Representative *in vivo* calcium imaging of beta cells of *pnpo*$^{+/+}$ zebrafish in the *Tg(ins:GCaMP6s); Tg(ins:cdt1-mCherry)* background. The movie displays 3 consecutive recordings of the same zebrafish with glucose stimulations. The first 10 s in each recording corresponds to the baseline calcium activity. The glucose stimulation is performed at 10 s, and the activity is recorded for 2 min. Scale bar = 10 µm

Movie S21. Calcium imaging of pnpo mutant (*pnpo*$^{-/-}$) zebrafish pancreatic islet. Representative *in vivo* calcium imaging of beta cells in *pnpo*$^{-/-}$ zebrafish in *Tg(ins:GCaMP6s); Tg(ins:cdt1-mCherry)* background. The movie displays 3 consecutive recordings of the same zebrafish with glucose stimulations. The first 10 s in each recording corresponds to the baseline calcium activity. The glucose stimulation is performed at 10 s, and the activity is recorded for 2 min. Scale bar = 10 µm

Movie S22. *In vitro* 1 calcium imaging of DMSO-treated islets from transgenic C57BL/6J Ins1Cre:GCaMP6f$^{fl/fl}$ mice. The first 3 minutes correspond to low glucose (3mM), followed by

33 min of imaging at 16 mM glucose. During the last 3 minutes, KCl is added to force membrane depolarization.

Movie S23. *In vitro* calcium imaging of 1mM 4-Deoxypyridoxine (4-DP)-treated islets from transgenic C57BL/6J Ins1Cre:GCaMP6f$^{fl/fl}$ mice. The first 3 minutes correspond to low glucose (3mM), followed by 33 minutes of imaging at 16 mM glucose. During the last 3 minutes, KCl is added to force membrane depolarization.

Data S1. (separate file)
List of differentially expressed genes based on CaMPARI and subsequent pathway analysis.

Data S2. (separate file)
List of differentially expressed genes between pnpo^{+} β and pnpo^{-} β-cells in zebrafish and human and subsequent pathway analysis.
